# Self-Reported Periodization of Nutrition in Elite Female and Male Runners and Race Walkers

**DOI:** 10.3389/fphys.2018.01732

**Published:** 2018-12-03

**Authors:** Ida Aliisa Heikura, Trent Stellingwerff, Louise Mary Burke

**Affiliations:** ^1^Mary MacKillop Institute for Health Research, Australian Catholic University, Melbourne, VIC, Australia; ^2^Sports Nutrition, Australian Institute of Sport, Canberra, ACT, Australia; ^3^Canadian Sport Institute Pacific, Victoria, BC, Canada

**Keywords:** nutrition periodization, elite athletes, endurance athletes, carbohydrate availability, questionnaire

## Abstract

Athletes should achieve event-specific physiological requirements through careful periodization of training, underpinned by individualized and targeted nutrition strategies. However, evidence of whether, and how, elite endurance athletes periodize nutrition is scarce. Accordingly, elite international female (*n* = 67) and male (*n* = 37) middle/long-distance athletes (IAAF score: 1129 ± 54, corresponds to 13:22.49 [males] and 15:17.93 [females] in the 5000 m) completed an online survey (February–May 2018) examining self-reported practices of dietary periodization for micro (within/between-days), meso (weeks/months) and macro (across the year) contexts. Data are shown as the percentage of all athletes practicing a given strategy followed by the % of athletes reporting various beliefs or practices within this strategy. Differences according to sex, event (middle-distance [800 m/1500 m] vs. track-distance [3000 m-10000 m] vs. road-distance [marathon/race walks]), caliber (high [major championship qualifier] vs. lower), and training volume (low/moderate/high male and female tertiles) were analyzed using Chi-square test or Kruskal–Wallis Test and indicated statistically different when *p* ≤ 0.05. Most athletes reported eating more on hard training days (92%) and focusing on nutrition before (84%; carbohydrate intake [63%] and timing [58%]) and after (95%; protein goals [59%], timing [55%], carbohydrate goals [50%]) key sessions. Road-distance were the most (62 and 57%), and middle-distance the least (30 and 30%) likely to train fasted (*p* = 0.037) or restrict carbohydrates periodically (*p* = 0.050), respectively. Carbohydrate intake during training (58% of total) was more common in males (79%; *p* = 0.004) and road-distance (90%; *p* < 0.001) than females (53%) or middle/track-distance (48 and 37%). Most athletes (83%) reported following a specific diet before and during race day, with half of the athletes focusing on carbohydrates. Nearly all (97%) road-distance athletes reported following a during-race nutrition plan (carbohydrates/fluids:89%). Only 32% reported taking advice from a dietitian/nutritionist. Based on our analysis: (1) Road-distance athletes periodize carbohydrate availability while track/middle-distance avoid low carbohydrate availability; (2) Middle-distance runners emphasize physique goals to guide their nutrition strategies; (3) Females seem to be more cautious of increasing energy/carbohydrate intake; (4) Among all athletes, nutrition strategies are chosen primarily to improve performance, followed by reasons related to physique, adaptation and health outcomes. Overall, these athletes appear to possess good knowledge of nutrition for supporting training and competition performance.

## Introduction

Despite decades of interest in the periodization of training, it is only recently that a holistic approach to periodization across a range of themes that affect competition preparation has been suggested (Burke et al., 2018; Mujika et al., 2018; Stellingwerff et al., 2018). In fact, the concept of integrating a periodized nutrition plan within the annual training program was formally proposed in a previous expert panel around nutrition for track and field athletes by Stellingwerff et al. (2007). The principles, practices and terminology around the periodization of nutrition have been summarized in several recent reviews (Jeukendrup, 2017; Burke et al., 2018; Stellingwerff et al., 2018). The underlying theme is that strategic and targeted nutritional interventions can be used to augment the outcomes of the various specific training cycles [micro (within-day to days), meso (several weeks) and macro (months to years)]. Thus, decisions on periodization of nutrition should be preceded by a thorough examination and understanding of the sport-specific (general) determinants of success as well as athlete-specific (individual) performance gaps, with strategies (including nutrition interventions) to address the gaps being integrated into the periodized training program (Stellingwerff et al., 2018).

A variety of aspects of nutrition can be periodized in support of different training goals, ranging from fundamental issues such as energy intake through to the more specialized and “fine tuning” aspects of supplement use (Jeukendrup, 2017; Stellingwerff et al., 2018). From a macro perspective, energy intake needs to be manipulated across, and within, training days according to fluctuations in the energy cost of the athlete’s training program, as well as strategic integration of periods of alterations to energy balance to manipulate body mass/composition (Melin et al., 2018; Stellingwerff, 2018). Here, it is important to recognize that energy mismatches due to deliberate efforts to reduce body mass/fat content, or the failure to account for the energy cost of a heavy training loads for prolonged periods are likely to impair health and performance with both short- and long-term consequences (Mountjoy et al., 2018). At the other end of the spectrum, periodized use of supplements may range from the use of iron supplements to ensure adequate iron status during altitude training block (Mujika et al., 2018), to the use of performance aids such as caffeine, creatine, buffers (e.g., beta-alanine or bicarbonate) and nitrate/beetroot juice to practice intended competition strategies, or to provide support for targeted training sessions.

The periodization of macronutrients includes themes of meeting the specific fuel needs of training and competition sessions (micro-periodization: particularly in the case of carbohydrate [CHO], and perhaps, fat intake), practicing event nutrition strategies (meso-periodization: particularly CHO intake during longer events) and providing both an additional stimulus and the building blocks needed to optimize the synthesis of new proteins as part of the adaptation to training (micro-, meso-, and/or macro-periodization: e.g., protein intake). Current guidelines around protein intake for all types of athletes promote the regular intake of modest amounts (e.g., ∼25 g every 3–4 h) of high quality protein over the day, including soon after the completion of key training sessions (i.e., sessions of high intensity and/or high duration [>90 min]) (Phillips and Van Loon, 2011). Meanwhile, there may be advantages in increasing protein intake during periods of deliberate energy manipulation to achieve loss of body fat to assist with the maintenance of lean mass (Hector and Phillips, 2018). The amount and timing of CHO intake between and within days should track the substrate needs of training and events (Areta and Hopkins, 2018), particularly when it is important to optimize performance in competitions or key training sessions (Thomas et al., 2016). There may also be a need to undertake specific strategies to train the gut to tolerate increasing amounts of CHO and fluids during exercise (Cox et al., 2010; Jeukendrup, 2014), in preparation for race nutrition practices during prolonged events, particularly in hot environments, where intake during the event plays a major role in performance success. Taken together, there are numerous examples of macro-, meso-, and micro-periodization of nutrition to optimize training adaptation and/or acute sport performance.

Although CHO intake is mostly considered in relation to its role as a key fuel for the muscle and brain, the application of molecular techniques to investigate the muscle response to exercise has created interest in the effects of low CHO availability on cellular signaling and enhanced adaptation to endurance training. According to various reviews (Philp et al., 2011; Bartlett et al., 2015; Hearris et al., 2018; Impey et al., 2018), undertaking endurance exercise with an environment of low CHO availability during and/or after the session may upregulate the activity of key molecules in the adaptive responses to exercise, leading to an enhanced or prolonged adaptation period. Observations of increases in the acute response to exercise have led to studies of the chronic implementation of periodized CHO availability (i.e., integration of strategies of high CHO availability to “train hard” for optimized performance, and strategies of low CHO availability to “train smart” with enhanced adaptation) to test its effect on performance outcomes. It is important to note that it can be difficult to achieve the right balance between training quality and adaptation within a controlled laboratory study design (Yeo et al., 2008; Hulston et al., 2010). Accordingly, although a few studies have reported superior performance outcomes in cohorts of trained/well-trained individuals (Marquet et al., 2016a,b), the translation to elite athletes seems more difficult (Burke et al., 2017; Gejl et al., 2017). Nevertheless, the strategy has been integrated by some elite athletes (Stellingwerff, 2012) and recognized as an emerging concept in the most recent sports nutrition guidelines (Thomas et al., 2016). One challenge for athletes, coaches and sports scientists is understanding the meanings and nuances of different strategies to periodize CHO availability within training and competition preparation. However, this has been addressed in a recent commentary in which terminology, practices, mechanisms and evidence of different strategies have been summarized (Burke et al., 2018).

In recognizing the value of a periodized approach to sports nutrition, current guidelines also promote the importance of individualization, which is dependent upon the specific performance demands of the sport/event and the unique athlete response to the intervention. Factors that may influence the individualized implementation of a specific nutrition strategy might include: (1) race distance (e.g., middle-distance athletes may not benefit from training the gut to consume liquids, while this is an important strategy for most road athletes); (2) event specific body composition norms; (3) training/event volume (e.g., higher volume training may require more emphasis on adequate energy/fuel availability); (4) training location (e.g., altitude/heat); and (5) time of the year (e.g., protocols to improve body composition and race performance might be emphasized closer to the competition season) (Stellingwerff et al., 2007; Stellingwerff, 2012; Melin et al., 2018; Stellingwerff et al., 2018). Therefore, it is expected that each athlete will have a unique and constantly changing periodized nutrition plan suited to their specific needs.

In parallel to the growing evidence base for the value of a periodized approach to nutrition, there is interest in understanding whether/how elite athletes practice these strategies within the real-world annual training/racing calendar. The available studies are limited to endurance and team sports athletes who have provided a snapshot of the micro- and meso-periodization of nutrition during training phases (Burke et al., 2003; Bradley et al., 2015; Naughton et al., 2016; Heikura et al., 2017a; Anderson et al., 2017a,b) or around competition (Stellingwerff, 2012, 2018).

More recently, we completed preliminary work to characterize self-reported approaches to periodization of nutrition over the annual training plan (Heikura et al., 2017b). Our pilot project captured an account of practices and the underlying rationale for nutritional periodization across the year (macro-periodization), with special consideration of various micro (between/within-day) and meso (various training/competition phases) cycles in 48 elite distance and middle distance track and field athletes. Having tested and updated this pilot study survey, in the current study we embarked on the investigation of the self-reported practices of dietary periodization across the annual training/racing calendar in a large cohort of world-class track and field endurance athletes Our goal was to characterize periodized nutrition practices across the year (macro cycle), and during specific meso and micro cycles of training/racing in this group, with attention to the effects of sex, athlete caliber, event duration and volume of training on these practices.

## Materials and Methods

### Study Design and Participants

Based on our pilot study using a similar survey self-reported approach (Heikura et al., 2017b) we further developed the current study’s survey into an online tool consisting of variously themed questions around dietary micro-, meso-, and macro-periodization across the various annual training phases. Along with strategic changes to the survey, gained from insights from our pilot study, we also aimed for this be completed by a larger (target = 100) and more internationally representative group of athletes. We recruited elite female and male middle/distance athletes using online advertisements as well as direct contacts (via email or word-of-mouth) to athletes, coaches, applied sports practitioners and national sporting organizations (in Canada, United States, Australia, Japan, and Finland). To be included in the study, the athletes needed to be ≥ 18 years of age, currently and actively racing in the middle (800 m, 1500 m), distance (3000–10000 m) or road (half-marathon/marathon, 20 k/50 km race walk) events under the International Association of Athletics Federations (IAAF) and have a personal best of ≥ 1043 IAAF points (this corresponds to 5000 m time of 13:47.26 and 16:03.84 in males and females, respectively). Recruitment and completion of the surveys were completed between February 8 and May 21, 2018. The Ethics Committee of Australian Catholic University approved the study protocol which conformed to the Declaration of Helsinki.

### The Survey

The survey consisted of an updated version of our pilot study based on our reflections on the responses from the original cohort and additional feedback from colleagues and athletes. Whereas the pilot survey included a total of 29 questions (7 main questions and 22 sub-questions), the updated survey was expanded to include a total of 59 questions (19 questions on training/racing characteristics, plus 13 main and 27 sub-questions around nutritional practices). The final version of the survey was built online using SurveyGizmo (Boulder, CO, United States). Skip logic was used, building a custom path through the questions according to the respondent’s answers, for an improved participation experience (less confusion) and efficiency (less time to complete the survey).

The survey was completed anonymously, and an informed consent (a prerequisite for completing the survey) was completed as part of the online survey by all participants. The first part of the survey included background information, instructions, and general subject information. Thereafter, the athlete was asked to choose one of the two annual training periodization programs (track [e.g., 800–10000 m]) vs. road [e.g., the marathon and race walks] that best reflected his/her yearly program. Questions approached annual (macro) periodization of nutrition as a whole (*Part A: general principles of annual training/competition diet*) and as typically defined separate periodized training phases (meso cycles): *Part B: Base / endurance training phase*, *Part C: Main competition season* (i.e., several months in duration: track athletes) or *preparation for competition* (i.e., one or more weeks in duration: road athletes), and *Part D: Nutrition immediately before and on race day*. Part A also included questions on general nutrition principles (e.g., vegetarian, paleo, very high energy, low carb high fat, gluten free) in the overall diet or during specific time periods (e.g., altitude training or during return from illness/injury). Additionally, parts B, C, and D asked questions on training volume, key session and race frequency as well as number of race peaks. Across the survey and throughout this manuscript, hard training days were defined as “high volume and/or intensity days” and key sessions as “high intensity and/or high duration [ > 90 min] sessions or serious gym sessions.” Fueling was defined as eating foods (CHO foods, protein foods, sports foods, etc.) before training. Fasted training was defined as “training first thing in the morning without having eaten any food or consumed any other carbohydrates, or training later in the day without having eaten any carbohydrates for at least 8 h prior.” Within the survey, reminders of these terminology were included within each question that targeted nutrition in relation to these themes. Finally, the survey ended with an open, but optional, comment box.

It is important to note that the survey was purposely constructed to apply to the culture, practices and terminology used in endurance events in track and field. As such it is not directly applicable to other sports, and if used for other populations, even among endurance sports, it will need to be customized to the specific characteristics of these sports. A sample survey has been provided in the Supplementary Material to this paper.

### Data Management and Statistical Analysis

The data were checked and cleaned by excluding duplicate responses (i.e., two responses from the same individual), responses that were clearly false or confusing, and responses from those that did not satisfy the requirement of ≥1043 IAAF points. The answers were classified into clusters for further analysis using groupings based on *sex* (male vs. female), *distance* (middle distance (MidD; 800, 1500 m) vs. track distance (TrackD; 3,000 m steeplechase to 10,000 m) vs. road distance (RoadD; marathon, race walks)), *sex-based training volume groupings* based on within sex tertiles of the entire data set (Females: low: ≤100 km/wk, moderate: 101–129 km/wk, high ≥130 km/wk; Males: low: ≤119 km/wk, moderate: 120–155 km/wk, high ≥156 km/wk) and athlete *caliber* (High: major championship (Olympics or World Championships) medalist, finalist and/or qualifier; Lower: the rest of the data set).

Data were first organized using Microsoft Excel, while further statistical analyses were conducted using SPSS Statistics 22 software (INM, Armonk, NY, United States). Data are presented as means ± standard deviations (SD) and number (n) and percentage (%) of responses. Normality of continuous data was checked with the Shapiro–Wilk goodness-of-fit test. Student’s t-test for independent samples was used to test for differences in age, training volume and IAAF scores between subgroups. For YES/NO answers, % was calculated from the n of the total sample; for sub-questions, % was calculated from the remaining n resulting after the main initial question. Chi-square test (X^2^) for independence with Yates Continuity Correction along with phi effect size statistic were used to test for differences between subgroups. Where more than two subgroups were present, Kruskal–Wallis Test was used as a post hoc test. Across the paper, statistical significance is shown when *p* ≤ 0.05. In addition to numerical outcomes, relevant quotes provided by athletes (in cursive) have been embedded in the results section to provide further qualitative insights into the topic in question. To aid in the interpretation of results from the lengthy survey, aggregation of consistent outcomes across the survey was undertaken by all authors upon visual inspection of figures to develop a series of themes.

## Results

### General Questionnaire Outcomes

A total of 104 athletes (67 female and 37 male) from middle (*n* = 27), distance (*n* = 34), and road (*n* = 43) groups were included in the final analysis. Fifty-two were classified as high and 52 as lower caliber. Training volume groupings resulted in 33, 31 and 31 athletes classified as low, moderate and high volume, respectively. The majority of responses came from athletes born in United States (25%), Canada (21%), and Japan (13%), while the rest were from Australia and New Zealand (12%), the Nordic countries (10%), Western Europe (14%), South America (4%), and Africa (1%). Training/competition phase specific training and racing characteristics for all athletes pooled, and for specific sex- and event-subgroups, are shown in Table 1. It is worth noting that while the difference in training volume was significant between males and females, this difference is likely to disappear if training volume were to be assessed by minutes of total training. For example, most elite males complete training at around 3:30 min/km pace while most elite females complete their training at around 4 min/km pace. Therefore, if this assumption was adjusted for in the results, the total training time for males (∼472 min/week) and females (∼468 min/week) would be almost equal. MidD and TrackD athletes showed meso-periodization of training volumes, whereby training volume was significantly less during the competition season compared to the base training phase (Table 1). This variation in training load was absent among RoaD, who reported equal training volumes between base training and preparation for competition. However it should be noted that for RoadD athletes, preparation for competition included the ∼8 weeks before a key race, where training volumes might be maintained at a relatively high levels until ∼2 weeks before the race. This is different to track athletes, whose competition season may be extended over several seeks/months (10–11 weeks; Table 1) and where the athlete is likely to take part in frequent racing across this time period.

**Table 1:** Participant background and characteristics of training and competition in elite middle/long-distance athletes (all, sex-based comparisons, distance-based comparisons).

	All (*n* = 104)	Sex comparison		Distance comparison		
		
		Females (*n* = 67)	Males (*n* = 37)	MidD (*n* = 27)	TrackD (*n* = 34)	RoadD (*n* = 43)
Age (year)	29.2 ± 5.8	28.9 ± 5.8	29.6 ± 5.9	26.5 ± 3.6^∗∗∗^	28.3 ± 6.3 #	31.6 ± 5.7
IAAF score	1129 ± 54	1135 ± 54	1119 ± 54	1135 ± 48	1119 ± 45	1133 ± 64
Training/competition background (years)	9.4 ± 4.6	8.5 ± 3.5Ł	11.2 ± 5.8	7.1 ± 3.2^∗∗∗^	9.3 ± 3.6	11.0 ± 5.4
***Base training phase***
Training volume (km/wk)	123 ± 39	117 ± 39Ł	135 ± 36	92 ± 23^∗∗∗^	126 ± 41$	139 ± 34
Number of key sessions per week	3.4 ± 1.3	3.3 ± 1.4	3.4 ± 1.2	3.2 ± 1.0	3.2 ± 1.0	3.6 ± 1.7
***Competition season***
Season length (weeks)	9.3 ± 3.8	9.3 ± 3.7	9.5 ± 4.2	10.9 ± 1.6^∗∗∗^	9.8 ± 3.8 ##	3.8 ± 3.3
Number of serious races within season	5.1 ± 2.2	5.1 ± 2.2	4.9 ± 2.6	5.6 ± 1.6^∗^	5.2 ± 2.6	3.0 ± 2.1
***Preparation for competition***
Season length (weeks)	4.5 ± 2.6	4.2 ± 2.7	4.9 ± 2.6	N/A	3.3 ± 1.8	5.1 ± 2.8
Number of peaks per year	2.0 ± 0.7	2.0 ± 0.6	2.0 ± 0.8	N/A	1.7 ± 0.9	2.1 ± 0.6
Number of serious races within a peak	1.7 ± 0.9	1.9 ± 0.9	1.5 ± 1.0	N/A	2.0 ± 1.3	1.6 ± 0.8
Training volume during competition season/ preparation for competition (km/wk)	115 ± 48	109 ± 52	123 ± 41	73 ± 19^∗∗∗^^aaa^	118 ± 46$$$ ^aaa^	137 ± 47


*Values are means ± standard deviations. IAAF score, International Association of Athletics Federations scoring tables 2017; MidD, middle distance (800, 1500 m); TrackD, track distance (3000 m steeplechase to 10000 m); RoadD, road distance (marathon, race walks). N/A, not applicable (i.e., these questions were not part of the survey for this population). £*p* < 0.05 significant difference between sexes; ^∗^*p* < 0.05, ^∗∗∗^*p* < 0.001 significant difference between MidD and RoadD; #*p* < 0.05, ##*p* < 0.01, ###*p* < 0.001 significant difference between TrackD and RoadD; $*p* < 0.05, $$*p* < 0.01, $$$*p* < 0.001 significant difference between MidD and TrackD; ^aaa^*p* < 0.001 significant difference compared to base training phase.*

**FIGURE 1:**
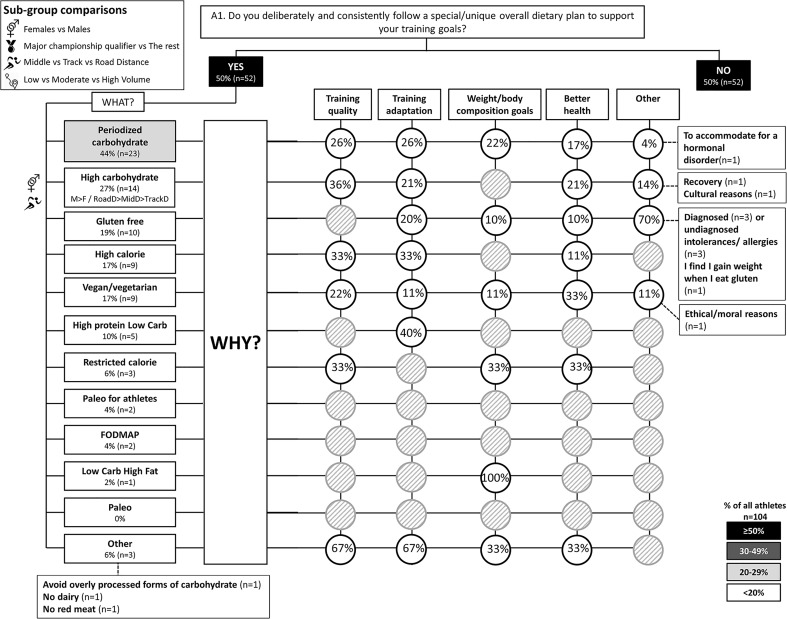
A1: Overall dietary practices across the year. The prevalence of specific, consistent nutrition practices (e.g., vegetarian) and the reasons for following them in 104 elite female and male track and field endurance athletes. Percentages (*%*) reflect the % of athletes that chose a specific answer in relation to all athletes (YES/NO answers) or in relation to specific sub-question populations (i.e., % of those that answered YES or NO). In addition, number (n) of athletes per each answer box has been provided. Answer boxes or circles are color coded based on % of all athletes as follows: ≥ 50%, *black box with white font*; 30–49%, *dark gray box with white font*; 20–29%, *light gray box with black font*; < 20%, *white box with black font*. Light gray circles with diagonal stripes indicate zero responses to this option. Symbols have been used to reflect significant (*p* < 0.05) between-group differences between sexes (*vector sex symbol*), athlete caliber (*medal symbol*), distance event (*runner symbol*), and reported volume during base training (*distance symbol*). Where significant differences were detected, answer boxes include a brief description of direction of difference, for example, M > F reflects a higher % of males (M) compared to females (F) for that answer. *MidD*, Middle Distance (800 and 1500 m); *TrackD*, track distance (3000 m steeplechase to 10,000 m); *RoadD*, road distance (marathon and race walks). High caliber, major championship qualifiers; Lower caliber, those that have not qualified to major championships. *Low, Moderate*, and *High Volume* groups as sex-specific tertile cut-offs.

**FIGURE 2:**
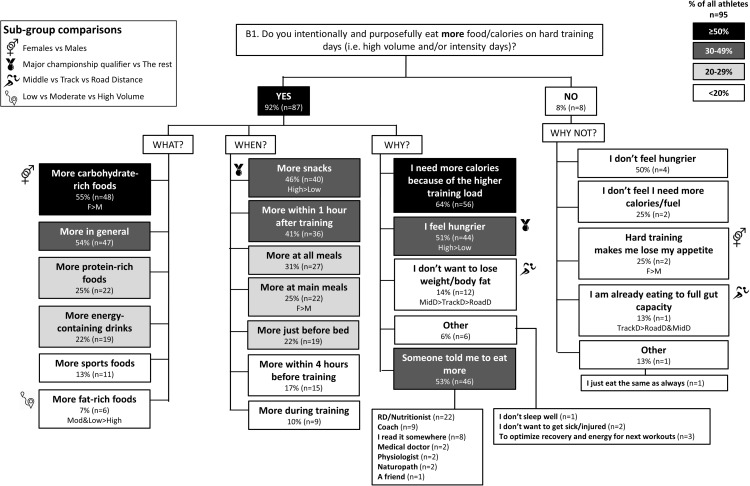
B1: Nutrition on hard training days during base training phase. The prevalence of specific nutrition practices on hard training days and the reasons for following them in 95 elite female and male track and field endurance athletes. Please see full description for figure within Figure 1.

**FIGURE 3:**
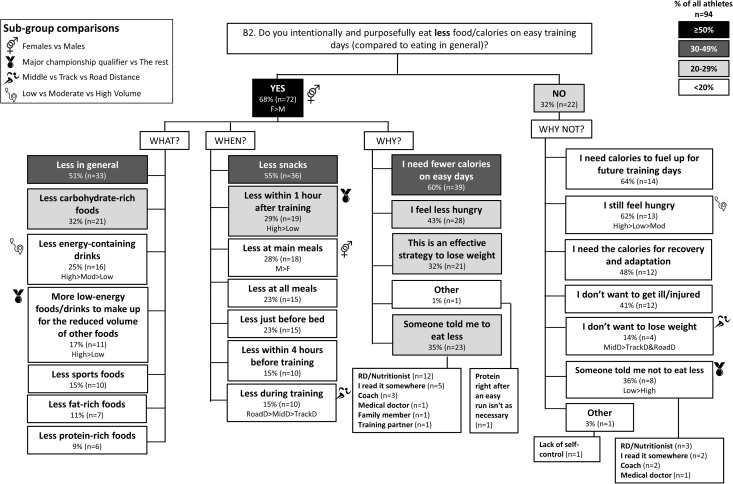
B2: Nutrition on easy training days during base training phase. The prevalence of specific nutrition practices on easy training days and the reasons for following them in 94 elite female and male track and field endurance athletes. Please see full description for figure within Figure 1.

**FIGURE 4:**
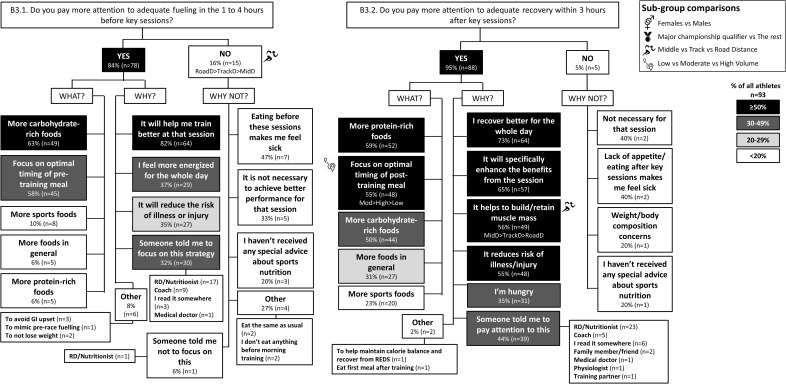
B3: Nutrition before (3.1) and after (3.2) key training sessions during base training phase. The prevalence of specific nutrition practices around fueling and recovery from key training sessions and the reasons for following them in 93 elite female and male track and field endurance athletes. Please see full description for figure within Figure 1.

**FIGURE 5:**
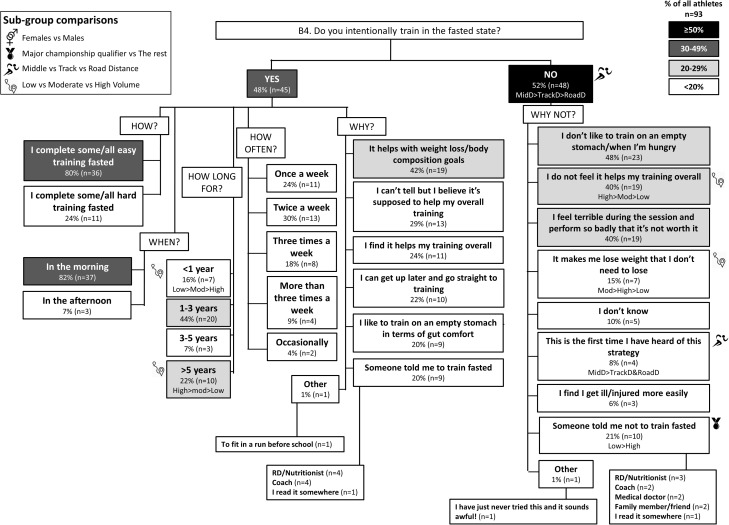
B4: Fasted training during base training phase. The prevalence of training in the fasted state with specific details around timing, frequency and reasons for this strategy in 93 elite female and male track and field endurance athletes. Please see full description for figure within Figure 1.

**FIGURE 6:**
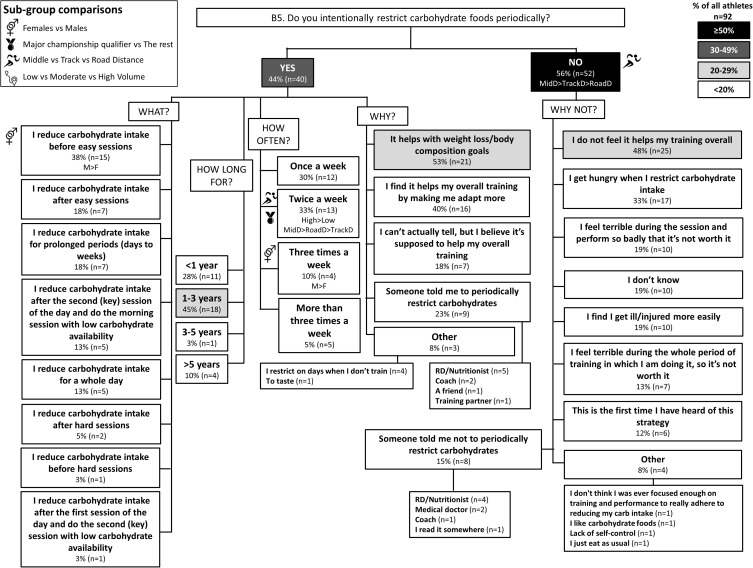
B5: Periodic carbohydrate restriction during base training phase. The prevalence of restricting carbohydrate intake periodically with specific details around timing, frequency and reasons for this strategy in 92 elite female and male track and field endurance athletes. Please see full description for figure within Figure 1.

**FIGURE 7:**
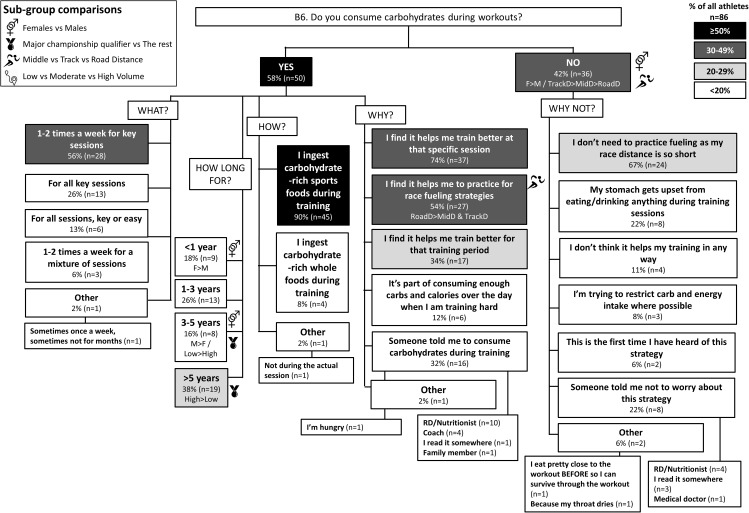
B6: Gut training during base training phase. The prevalence of training the gut (i.e., ingesting carbohydrates during workouts) with specific details around timing, frequency and reasons for this strategy in 86 elite female and male track and field endurance athletes. Please see full description for figure within Figure 1.

**FIGURE 8:**
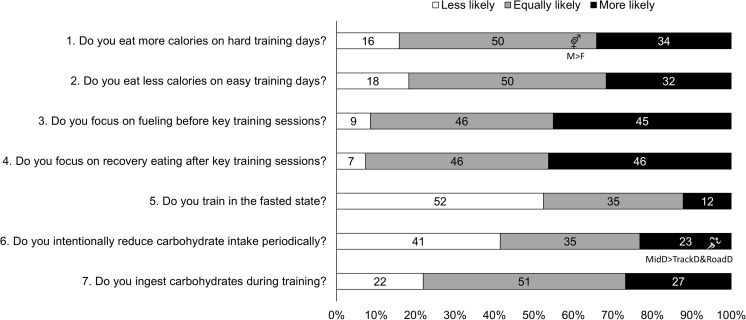
C: Major nutrition strategies implemented during competition season and preparation for competition as compared to base/endurance phase. Answers to Part C: “Compared to nutrition during base/endurance training phase, how much do you focus on the following dietary strategies during competition season (track athletes) or preparation for competition (road athletes)?” Values are percentages of all athletes (*n* = 83): *white bars*, less likely; *gray bars*, equally likely; *black bars*, more likely to follow this strategy. Symbols have been used to reflect significant (*p* < 0.05) between-group differences between sexes (*vector sex symbol*), and distance event (*runner symbol*). Where significant differences were detected, the symbols are combined with a brief description of direction of difference, for example, M > F reflects a higher % of males (M) compared to females (F) for that answer. *MidD*, Middle Distance (800 and 1500 m); *TrackD*, track distance (3000 m steeplechase to 10,000 m); *RoadD*, road distance (marathon and race walks).

**FIGURE 9:**
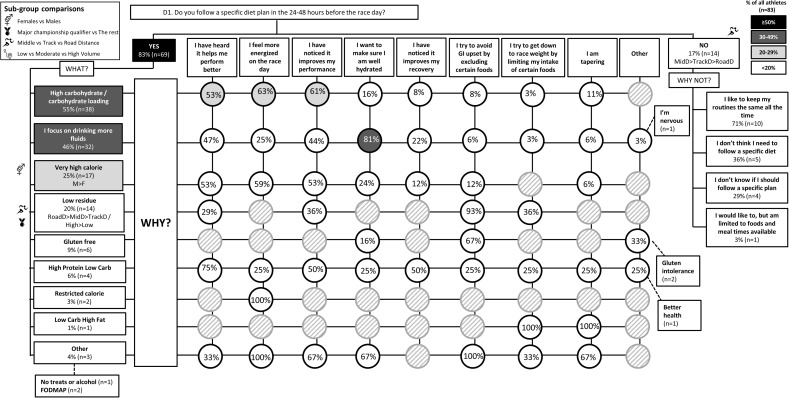
D1: Nutrition in the 24–48h time period before the race. The prevalence of specific nutrition strategies and the reasons for them in 83 elite female and male track and field endurance athletes within the acute time period preceding the main race. Please see full description for figure within Figure 1.

**FIGURE 10:**
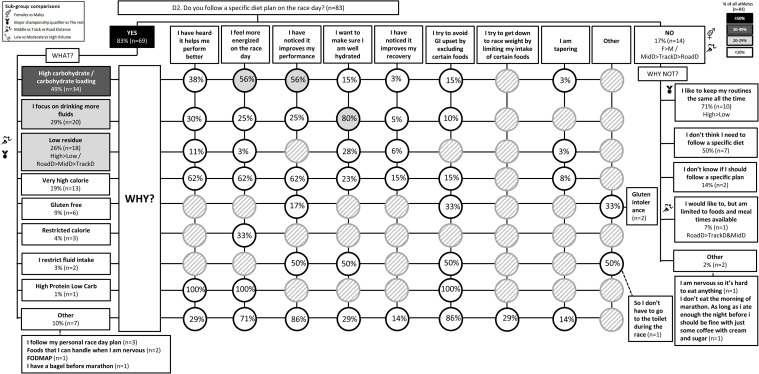
D2: Nutrition on race day. The prevalence of specific nutrition strategies and the reasons for them in 83 elite female and male track and field endurance athletes on the day of the main race. Please see full description for figure within Figure 1.

**FIGURE 11:**
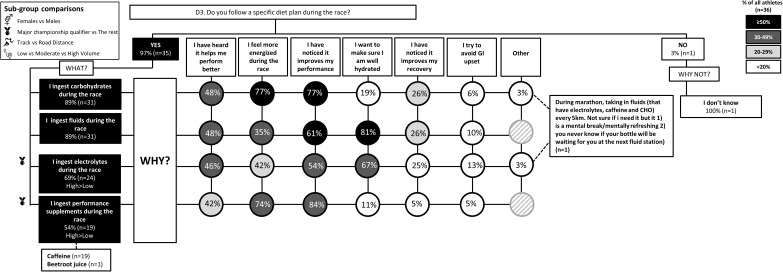
D3: Nutrition during the race. The prevalence of specific nutrition strategies and the reasons for them in 36 elite female and male track and field endurance athletes during the main race. Only athletes competing in road events replied to this question. Please see full description for figure within Figure 1.

The results of the survey are summarized in the following figures: Figure 1 (Overall dietary practices across the year); Figure 2 (Eating on hard training days); Figure 3 (Eating on easy training days); Figure 4 (Fueling and recovery around key sessions); Figure 5 (Training in the fasted state); Figure 6 (Periodic CHO restriction); Figure 7 (Ingestion of CHO during training); Figure 8 (Major nutrition strategies implemented during competition season or preparation for competition); Figure 9 (Nutrition in the 24–48 h before the race day); Figure 10 (Nutrition on the race day); Figure 11 (Nutrition during the race). Key themes that emerged from these data are now discussed.

### Theme 1: Road-Distance Athletes Utilize Much Greater Extremes of CHO Availability (Low to High) During Training Compared to Middle- or Track-Distance Athletes

A chronically high CHO diet was significantly more common among RoadD (46%) compared to TrackD [6%; *F*(2) = 10.195, *p* = 0.009] or MidD (10%) athletes (Figure 1). RoadD (62%) were also more likely to train fasted during base training compared to MidD (30%; *p* = 0.032) (Figure 5). Similarly, purposeful CHO restriction was more popular among RoadD (57%) compared to MidD (30%; *p* = 0.047) (Figure 6). In terms of training with high CHO availability, ingestion of CHO during workouts in the base training phase was more common among RoadD (90%) compared to TrackD (37%) or MidD (48%; *p* < 0.001), mainly to practice race fueling [22% MidD vs. 38% TrackD vs. 67% RoadD, *F*(2) = 6.534, *p* = 0.038] (Figure 7). A higher number of RoadD (97%) and TrackD (85%) compared to MidD (57%) reported following a special diet in the 24-48h period preceding the race (MidD vs. RoadD, *p* < 0.001; MidD vs. TrackD, p = 0.039) (Figure 9). During this time, a low residue diet (i.e., low in fiber and whole foods) was more popular among RoadD (34%) compared to TrackD [5%; *F*(2) = 8.546, *p* = 0.021] or MidD (8%). Most RoadD (97%) compared to two-thirds of MidD (67%; *p* = 0.009) reported to follow a special diet on race day (Figure 10).

### Theme 2: Middle-Distance Athletes Focus on Nutrition Strategies to Manipulate Physique

During the base training phase, MidD (32%) were more likely to report eating more on hard training days to prevent weight loss, compared to RoadD [5%; *F*(2) = 7.181, *p* = 0.022], with no difference to TrackD (13%) (Figure 2). Furthermore, compared to TrackD (0%) and RoadD (0%), MidD (40%) were more likely to avoid eating less on easy training days because they did not want to lose weight [*F*(2) = 8.512, *p* = 0.023] (Figure 3). MidD (80%) were also more likely than TrackD (52%) or RoadD (46%) to focus on eating after key sessions to help retain/build muscle mass [*F*(2) = 6.349, *p* = 0.042 between MidD and RoadD] (Figure 4). MidD included individuals (19%) that had never heard of fasted training before [compared to 0% of TrackD and RoadD; *F*(2) = 6.267, *p* = 0.044]. During the competition season, MidD were more likely to focus on CHO restriction compared to RoadD [38% vs. 9%, *F*(2) = 7.574, *p* = 0.023] (Figure 8).

### Theme 3: Females Are More Conscious of Intake of Extra Energy/CHO Than Males

A higher proportion of males than females [47% vs. 16%, *X*(1) = 5.287, *p* = 0.022] reported to follow a chronically high CHO diet (Figure 1). In addition, more females [79%; *X*(1) = 7.412, *p* = 0.006] than males (52%) reported eating less on easy days during the base training phase (Figure 3). CHO intake during training was more popular among males (79%) than females (53%; *p* = 0.004) (Figure 7). In the acute time period preceding the race day, more males than females reported following a high energy diet [36% vs. 15%; *X*(1) = 4.151, *p* = 0.042] (Figure 9). Also, 76% females vs. 94% males [*X*(1) = 4.187, *p* = 0.041] follow a special diet on race day (Figure 10). However it should be noted that 65% of males, compared to 29% of females, identified themselves as RoadD, which has most likely influenced the outcomes.

### Theme 4: Performance Is the Main Reason Behind Nutrition Strategies; Meanwhile Less Is Known About Nutrition for Adaptation

Overall, the quality of performance during training and racing seem to be the main driving factors behind the decision making when choosing a specific nutrition strategy. This theme is present in nutrition practices overall (Figure 1), as well as throughout specific micro, meso and macro levels of training and competition (Figures 2–11). Other common explanations for choosing a specific nutrition strategy were efforts to manipulate body composition (Figures 3–6), stay healthy/free of injuries (Figure 4), practicality (e.g., training before breakfast for time-management purposes; Figure 5), and because someone (usually a dietitian) told the athlete to do so. A substantial proportion of athletes seemed to be unaware of the usefulness of specific strategies to enhance training adaptation via periodically training in the fasted state and/or restricting CHO intake around training sessions (Figures 5, 6).

### Other Key Findings

Nearly half of the athletes (44%) reported following a periodized CHO diet over the annual training program (Figure 1), while ∼one fifth of the athletes followed gluten free diets (19%) and vegan/vegetarian diets (17%). Even less common were diets emphasizing low CHO intake such as Paleo (0%), low CHO high fat diet (2%), restricted calorie (6%) and high protein low CHO (10%).

Overall, most athletes (92%) reported eating more on hard training days (Figure 2), including more CHO-rich foods (55%) and more in general (54%), while 68% reported adjusting nutrition intake to match lower energy expenditure on easy training days (Figure 3), including less in general (51%) and fewer CHO-rich foods (32%). Nutritional strategies to prepare for key training sessions were important to most (84%) athletes with key themes of the choice of CHO-rich foods (63%) and timing (58%). Meanwhile, 95% of athletes prioritized recovery after these sessions with key themes of choosing protein-rich (59%) or CHO-rich (50%) foods or considering the timing of intake (55%) (Figure 4). Fasted training (48%; Figure 5) and periodic CHO restriction (44%; Figure 6) were practiced by almost half of the athlete cohort, with the main rationale being weight loss (42 and 53%, respectively). More than half of the athletes (58%) reported consuming CHO during workouts, with the focus on key sessions 1–2 times a week (56%) and to maintain training intensity (74%) (Figure 7). Competition nutrition strategies focused mainly on adequate CHO and fluid intake before (Figure 9) and on race day (Figures 10, 11) as well as on low residue diet throughout this time period. A number of athletes further explained their dietary choices, A selection of noteworthy athlete quotes are in Table 2.

**Table 2:** Selection of noteworthy athlete quotes regarding why they do, or do not, follow a specific nutrition strategy.

Nutrition strategy	Reason for following/not following this strategy
B3: Why do you focus on pre-key session fueling?	“*To help maintain calorie balance and recover from REDS*.”
B4: Why do you not train in the fasted state?	“*That will put me so far behind in terms of energy intake which I can’t afford.” “I’m very lean to begin with - I don’t think I’d make it through!” “I do not believe this approach is scientifically valid*.”
B5: Why do you restrict carbohydrate intake periodically?	“*I think there is something to making your body more insulin sensitive*.” “*It seems like a bad idea. My understanding is that the body uses carbs as primary fuel source.”*
B1: Why do you eat more food/calories on hard training days?	“*I feel like I have earned it*.”
B5: Why do you not restrict carbohydrate intake periodically?	“*I like carbohydrate foods*.” “*Lack of self-control*.”
D1: Why do you focus on drinking fluids in the 24-8 h before the race day?	“*I am nervous*.”
D2: Why do you not follow a specific diet on the race day?	“*I am nervous so it’s hard to eat anything*.”


### Sources of Information

One third (32%) of all athletes relied on a sports dietitian/nutritionist for nutrition advice. Of these, nearly half (43%) were MidD, while only 37% of TrackD and 20% of RoadD relied on this source of information [*X*(1) = 9.751, *p* = 0.008 between MidD and RoadD]. Other sources of information were less popular and included: coach (15%), read it somewhere (13%), training partner/a friend (5%), medical doctor (4%), physiologist (2%), naturopath (2%), and family member (1%), with no meaningful differences between subgroups.

## Discussion

This study aimed to characterize self-reported dietary periodization across macro (general practices across the annual cycle), meso (training and racing phases) and micro (between- and within-day) phases of training and competition in a large cohort of elite female and male middle- and long-distance runners and race walkers. We detected a number of key repeated themes across various levels of training periodization, including: (1) Road athletes reported different nutritional practices to middle- and track-distance athletes, by including strategies of training with both low and high CHO availability within the annual training plan; (2) Middle-distance athletes were the most conscious about the effects of nutrition strategies on physique outcomes; (3) Females seemed to be more conscious of intake of extra energy/CHO compared to males; (4) Overall, training and race performance appeared key factors influencing nutrition choices, while themes such as body composition manipulation, health, and practicality were less important; (5) Many athletes within this cohort of high level athletes were unaware of the use of nutrition to manipulate training adaptations, or felt that there were side-effects or challenges that prevented their use.

### Theme 1: Road-Distance Athletes Periodize CHO Availability Across the Year

Historically, nutrition guidelines for endurance athletes have focused on strategies to habitually achieve high CHO availability to support performance and recovery around training and races (Coyle, 1991). Protocols that supply sufficient CHO fuels to meet the demands of prolonged and/or high-intensity endurance sessions, such as consuming sufficient CHO to refuel glycogen stores prior to an event, including CHO loading for events > 90 min (Hawley et al., 1997), a CHO rich meal in the hours before exercise (Coyle, 1991) or CHO intake during prolonged exercise according to the duration and mode of exercise (Stellingwerff and Cox, 2014) can enhance performance by ∼2–3%. Contemporary recommendations support high CHO availability for competition, as well as for key training sessions in the athlete’s program in which high-intensity performance needs to be completed at the highest quality possible, or in which race nutrition strategies need to be practiced. In the current study, 90% of road-distance athletes (marathon runners and race walkers) reported strategies of ingesting CHO during workouts in the base/endurance training phase (Figure 7), while 89% focus on CHO intake during racing (Figure 11). Indeed, it seems that this cohort of elite road-distance athletes are aware of, and aim to, follow current sports nutrition guidelines that emphasize optimal CHO intake around key training and racing (Thomas et al., 2016). On the contrary, and as expected, these strategies were less important for athletes competing in shorter distance events where endogenous CHO fuel stores are not limiting.

Meanwhile, more recent studies have focused on the adaptation and performance effects of strategically and periodically implemented low CHO availability before, during, or after exercise (Bartlett et al., 2015; Hearris et al., 2018; Impey et al., 2018). These studies suggest that occasional and strategic training with low CHO availability increases the cell signaling and gene expression responses that are usually seen after endurance training, thereby leading to further enhanced endurance capacity and performance. Possible strategies, as detailed in a recent commentary of definitions and proposed outcomes (Burke et al., 2018) include fasted training (Figure 5), CHO restriction between the first and the second session of the day (Figure 6), CHO restriction during prolonged exercise, and CHO-restricted recovery overnight. While these strategies and their potential outcomes are intriguing, studies in elite athletes have failed to show direct performance benefits (Burke et al., 2017; Gejl et al., 2017). Furthermore, studies on bone and iron health suggest these strategies may impair bone and iron metabolism, possibly leading into increased bone breakdown (Sale et al., 2015) and decreased iron levels (Badenhorst et al., 2015). Therefore, careful day-to-day periodization is likely required, where low CHO availability is primarily scheduled around low intensity sessions (Hearris et al., 2018). In the current study, 62% of road-distance athletes reported undertaking some training sessions in a fasted state, while this strategy was only half as popular among middle-distance athletes (30%; Figure 5). Weight loss was the most popular reason for training fasted (42% of athletes), while only 29% of those who practiced this believed that it might help with training adaptations.

### Theme 2: Middle-Distance Athletes Focus on Optimal Physique

A more recent advancement in the field is periodization of body composition (Stellingwerff, 2018), which refers to the manipulation of body composition (via a mixture of nutrition and training strategies) for optimal health and performance. The underlying idea is that race weight should not be maintained year-round, as this is likely to require chronic periods of low energy availability (EA) and its related impairments of several health and performance related measures (Mountjoy et al., 2018). Therefore, EA may need to be periodized across the year, with emphasis on higher EA levels during heavy training and altitude camps, and lower EA during lower training volumes and closer to the competition season. In addition to this macro and meso periodization of EA, emerging evidence suggests that within-day EA (micro level periodization) has also significant health consequences (Deutz et al., 2000; Fahrenholtz et al., 2018; Torstveit et al., 2018). Indeed, timing of energy intake around exercise (as opposed to “backend loading” with the majority of energy intake consumed in the evening) may be a powerful tool to manipulate physique while maintaining health. In the current study, middle-distance athletes reported more attention to the effects of nutrition strategies on physique outcomes; however, their chief focus was to build and maintain lean mass. For example, 40% of middle-distance athletes reported a maintenance of their food intake on easy training days to avoid weight loss (Figure 3). In addition, these athletes focused on nutritional support immediately after key workouts to maintain/build muscle mass (Figure 4). During the competition season, however, middle-distance athletes were more likely to report a reduction in CHO intake or use of CHO restriction strategies (Figure 8), which may reflect a relative reduction in training volume and/or their efforts to reduce body mass to achieve an optimal race weight.

### Theme 3: Females Are More Conscious of Intake of Extra Energy/CHO Than Males

Females and males have an equal ability for CHO storage and utilization during exercise if energy availability is adequate (Tarnopolsky et al., 2001; Wallis et al., 2006). However, female distance athletes tend to eat less CHO than males (Burke et al., 2001), although this difference is likely to disappear when CHO intake is adjusted to training volume (Heikura et al., 2017a), as recommended by current guidelines (Thomas et al., 2016). Regardless of equal (relative) energy and CHO needs for female and male athletes, dietary practices of females tend to be more cautious of extra energy/CHO intake. Indeed, females are more likely to suffer from eating disorders (Sundgot-Borgen and Torstveit, 2004). This may be due to a higher frequency of body image issues/concerns over body weight among female athletes (Martinsen et al., 2010). In the current study, we showed similar patterns of calorie/CHO awareness among elite distance athletes as has been previously reported in sub-elite athlete populations. Namely, male athletes were more likely to follow a chronically high CHO diet (Figure 1). In addition, a greater proportion of females (79%) than males (52%) reported eating less on easy days during the base training phase (Figure 3). Males were also more likely to follow a high energy diet in the acute time period preceding the race day (Figure 9). Although qualitative, these outcomes suggest that female athletes may indeed be more concerned about consuming extra energy/CHO however whether the reasons are justified due to a lower fuel requirement, or related to eating disorders/disordered eating, lack of knowledge, or other factors, cannot be concluded based on the current survey.

### Theme 4: Nutrition Strategies Are Based on Performance Rather Than Adaptation

Contrary to previous guidelines (Coyle, 1991), more recent sports nutrition guidelines have incorporated the value of specialized strategies to optimize adaptations to training, noting that these protocols may often be contradictory for acute performance outcomes or other health goals, and need to be carefully integrated into the various phases of the annual plan (Thomas et al., 2016). We were interested to identify whether these concepts were understood by elite athletes and used to inform their various nutrition strategies. Our results suggest that the most common nutrition strategies reported by this large cohort (*n* = 104) of elite track and field distance athletes (of whom 50% were qualifiers for World Championships and/or Olympic Games) were focused on performance enhancement during training (Figures 2–4, 7) and competition (Figures 9–11). Meanwhile less was known about specific strategies to further stimulate cellular adaptations to exercise (Figures 5, 6). Indeed, many athletes lacked understanding of the periodization of strategies to train with low CHO availability, furthermore, others were either skeptical of their value, concerned about perceived or actual disadvantages particularly related to illness or injury, or practicing some aspects within their routines by accident. Since several outcomes identified the interest in using nutrition to manipulate body composition and/or to prevent illness/injuries, we conclude that the general priority for decisions around nutrition was performance > health > enhanced adaptation.

### Limitations

It is important to note that the current study describes self-reported nutrition practices that are implemented across the training and competition year. We have previously shown that there is a discrepancy between general descriptions of practices (reflecting a macrocycle) and actual self-recorded intakes (collected across a micro cycle) in elite distance athletes (Heikura et al., 2017a). Indeed, it is possible that self-reports such as those found in the current study, reflect either what athletes aspire to achieve or perceive that they follow rather than actual behaviors. However, this potentially perceived versus actual mismatch would hypothetically be equivalent across the various sub-groups of athletes. Furthermore, our survey questions were qualitative (i.e., describing “high” or “low” intakes instead of specific amounts) and it is possible that these relative terms are interpreted differently by different individuals. Nevertheless, our survey was based on the learnings from a pilot study (Heikura et al., 2017b) and we are confident that the expanded and improved survey tool had greater precision and sensitivity, along with more than 100 respondents, in detecting nutrition practices across all levels of training/racing periodization.

## Conclusion

We characterized self-reported dietary periodization across macro (general practices across the annual cycle), meso (training and racing phases) and micro (between- and within-day) phases of training and competition in 104 elite female and male middle- and long-distance runners and race walkers (50% major championship qualifiers). Our key findings suggest that: (1) Road athletes train with both low and high CHO availability within the annual training plan, while track athletes are less likely to incorporate a large spectrum of CHO availability in their training; (2) Middle-distance athletes emphasize physique when choosing a nutrition strategy; and (3) Performance appears to be the key driving factor influencing nutrition choices, while themes such as body composition manipulation, health, and practicality are less important. Overall, our findings indicate that elite track and field distance athletes are aware of and report following the current sports nutrition guidelines in terms of high CHO availability around key training sessions and during racing. However, most of this cohort appears to be unaware of and/or unwilling to aggressively incorporate the more recent strategies of training with reduced CHO availability to support training adaptations.

## Ethics Statement

This is a survey study which participants completed via an online survey tool. Consent to participate was completed via ticking in a box. The participants who proceeded to complete the survey were thus seen as consenting to participate in research.

## Author Contributions

IH, TS, and LB designed the study, developed the survey, recruited the participants, and prepared the manuscript. IH collected, organized, and analyzed the data. All authors approved the final manuscript.

## Conflict of Interest Statement

The authors declare that the research was conducted in the absence of any commercial or financial relationships that could be construed as a potential conflict of interest.
