# Supplementary material for: Self-Reported Periodization of Nutrition in Elite Female and Male Runners and Race Walkers
Source: Front Physiol. 2018 Dec 3;9:1732. doi: 10.3389/fphys.2018.01732 (PMC6286987; doi:10.3389/fphys.2018.01732)
Supplement: Supplementary file 1 [file Data_Sheet_1.PDF]

DIETARY PERIODIZATION ACROSS THE YEARLY TRAINING PROGRAM IN ELITE MIDDLE/DISTANCE TRACK AND FIELD ATHLETES

Consent to participate in the study

By ticking the box below and completing this questionnaire I sign the informed consent and thus approve the study design and the anonymous use of my responses for the study purposes by the research team.*

[ ] I have read and understood the information provided to me and give my consent to participate in research

PERIODIZATION OF ANNUAL TRAINING

1) Which of the following best describes your annual training/competition program (choose one)? (Note: if you are a road runner/race walker, your annual training program likely resembles option 2, and this cycle may be repeated twice if you have two peaks a year)*

( ) This model of periodization is usually typical of a track runner, whose competition season spans several months and includes multiple races

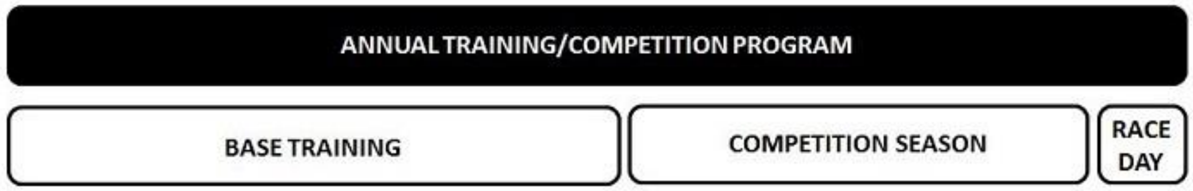

( ) This model of periodization is usually typical of a road runner/race walker, who peaks at a one particular time of the year for a key race

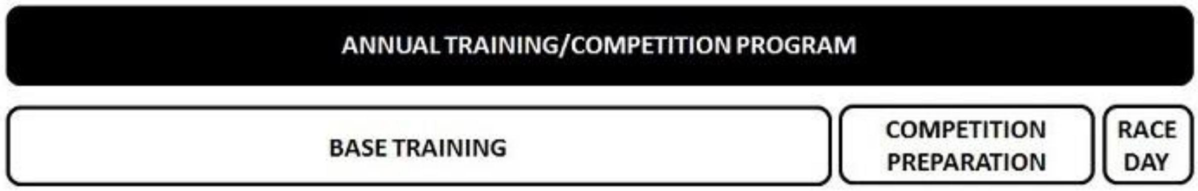

BACKGROUND INFORMATION

2) Gender*

( ) Female

( ) Male

3) Year of birth*

[dropdown list]

4) Country of birth*

[dropdown list]

5) Is the country of birth your country of residence (i.e. do you live in this country currently)?

( ) Yes

( ) No

6) If no, what is your country of residence?

[dropdown list]

7) Is the country of birth also the country you represent in your sport?

( ) Yes

( ) No

8) If no, what is your country of representation?

[dropdown list]

9) Primary event as a senior athlete*

( ) 800m

( ) 1500m

( ) 3000m steeplechase

( ) 5000m

( ) 10000m

( ) Marathon

( ) 20km race walk

( ) 50km race walk

10) Personal best in the primary event (in numbers)*

[minutes/seconds/hundreds of seconds]

11) Personal best in the primary event (in numbers)*

[hours/minutes/seconds]

12) Secondary event as a senior athlete (if applicable)

( ) 800m

( ) 1500m

( ) 3000m steeplechase

( ) 5000m

( ) 10000m

( ) Marathon

( ) 20km race walk

( ) 50km race walk

13) Personal best in the secondary event (in numbers)

[minutes/seconds/hundreds of seconds]

14) Personal best in the secondary event (in numbers)

[hours/minutes/seconds]

15) Self assessment of highest level of achievement as senior athlete*

( ) Medalist at the World Championships / Olympic Games

( ) Finalist at the World Championships / Olympic Games

( ) Qualifier at the World Championships / Olympic Games

( ) International level competitor (any competition that features several countries, such as the Diamond League or Pre Fontaine Classic)

( ) National Championship medalist

( ) National Championship participant

16)

Years of dedicated training/competition (give ranges if needed) **[This refers to your training background at a level where weekly training volume has been at least 8 hours/week, including weight training and ancillary training (recovery/yoga) and/or treatment]**

____

PART A: OVERALL NUTRITION PRINCIPLES ACROSS THE ANNUAL TRAINING/COMPETITION CYCLE

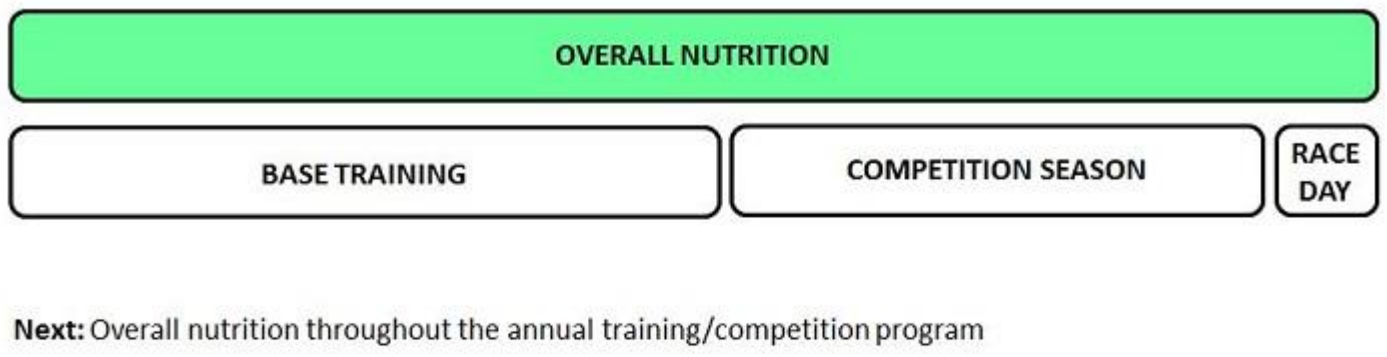

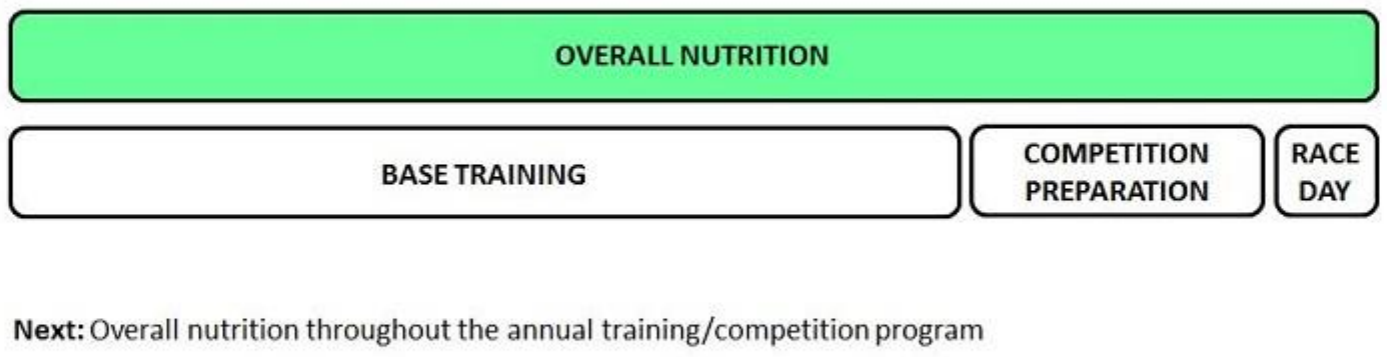

PART A. GENERAL PRINCIPLES OF YOUR ANNUAL TRAINING/COMPETITION DIET

17) Do you deliberately and consistently follow a special/unique overall general dietary plan (e.g. vegetarian) to support your training goals ? [i.e. all year round during all training phases] (Tick all the answers that are important in describing what you do)*

[ ] Vegan/vegetarian

[ ] Very high energy/extra energy (high Calorie)

[ ] Restricted energy (low energy)

[ ] Paleo

[ ] Paleo for athletes (Paleo but with more carbs around training sessions)

[ ] Low Carb High Fat (LCHF)

[ ] High protein Low Carb

[ ] Gluten Free

[ ] High Carb

[ ] Periodised Carb (strategic periodisation on carbohydrate intake around training, i.e. intentionally training with high or low carbohydrate availability)

[ ] FODMAP

[ ] Other (please describe): ____

[ ] No, I do not follow any special dietary plan

If yes, what is the main purpose of following a specific diet chronically? (Tick all the answers that are important in describing what you do)

	**Religious/cultural reasons**	**Ethical/moral reasons**	**To achieve better quality training overall**	**To enhance adaptation to training generally**	**To lose weight/achieve body composition goals**	**Better health**	**Diagnosed allergies or intolerances**	**I have some allergies or intolerances but haven't had them fully diagnosed**	**Other (please specify in the comment box below)**	**Someone told me to follow this diet (who? please specify in the comment box below)**
Vegan/vegetarian	[ ]	[ ]	[ ]	[ ]	[ ]	[ ]	[ ]	[ ]	[ ]	[ ]
Very high energy/extra energy	[ ]	[ ]	[ ]	[ ]	[ ]	[ ]	[ ]	[ ]	[ ]	[ ]
Restricted energy	[ ]	[ ]	[ ]	[ ]	[ ]	[ ]	[ ]	[ ]	[ ]	[ ]
Paleo	[ ]	[ ]	[ ]	[ ]	[ ]	[ ]	[ ]	[ ]	[ ]	[ ]
Paleo for athletes	[ ]	[ ]	[ ]	[ ]	[ ]	[ ]	[ ]	[ ]	[ ]	[ ]
Low Carb High Fat	[ ]	[ ]	[ ]	[ ]	[ ]	[ ]	[ ]	[ ]	[ ]	[ ]

High Protein Low Carb	[ ]	[ ]	[ ]	[ ]	[ ]	[ ]	[ ]	[ ]	[ ]	[ ]
Gluten free	[ ]	[ ]	[ ]	[ ]	[ ]	[ ]	[ ]	[ ]	[ ]	[ ]
High Carb	[ ]	[ ]	[ ]	[ ]	[ ]	[ ]	[ ]	[ ]	[ ]	[ ]
Periodised carb	[ ]	[ ]	[ ]	[ ]	[ ]	[ ]	[ ]	[ ]	[ ]	[ ]
FODMAP	[ ]	[ ]	[ ]	[ ]	[ ]	[ ]	[ ]	[ ]	[ ]	[ ]
Other	[ ]	[ ]	[ ]	[ ]	[ ]	[ ]	[ ]	[ ]	[ ]	[ ]

Comments:

PART A. GENERAL PRINCIPLES OF YOUR ANNUAL TRAINING/COMPETITION DIET

18) Do you deliberately but periodically introduce a special dietary plan to support your training goals? [i.e. periodically following one or several nutrition strategies over the annual training/competition program]
If so, when do you follow a special dietary plan? (Tick all the answers that are important in describing what you do)*

[ ] Altitude training

[ ] Heat training

[ ] Return from off-season

[ ] Return from injury

[ ] Competition preparation phase/transition to race season

[ ] Other time period (please explain): ____*

[ ] No, I do not periodically follow a special dietary plan during the yearly training program

What is the main purpose of following a special diet during altitude training? (Tick all the answers that are important in describing what you do)*

	**Cultural or religious reasons**	**Ethical/moral reasons**	**Better quality training for a special training block**	**Enhanced adaptation to a special training block**	**To lose weight/achieve body composition goals**	**Better health**	**Diagnosed allergies or intolerances**	**I have some allergies or intolerances but haven't had them fully diagnosed**	**Other (please explain)**
Vegan/vegetarian	[ ]	[ ]	[ ]	[ ]	[ ]	[ ]	[ ]	[ ]	[ ]
Very high energy/extra energy (high Calorie)	[ ]	[ ]	[ ]	[ ]	[ ]	[ ]	[ ]	[ ]	[ ]
Restricted energy (low energy)	[ ]	[ ]	[ ]	[ ]	[ ]	[ ]	[ ]	[ ]	[ ]
Paleo	[ ]	[ ]	[ ]	[ ]	[ ]	[ ]	[ ]	[ ]	[ ]
Paleo for athletes	[ ]	[ ]	[ ]	[ ]	[ ]	[ ]	[ ]	[ ]	[ ]
Low Carb High Fat (LCHF)	[ ]	[ ]	[ ]	[ ]	[ ]	[ ]	[ ]	[ ]	[ ]
High protein Low Carb	[ ]	[ ]	[ ]	[ ]	[ ]	[ ]	[ ]	[ ]	[ ]
Gluten Free	[ ]	[ ]	[ ]	[ ]	[ ]	[ ]	[ ]	[ ]	[ ]
High Carb	[ ]	[ ]	[ ]	[ ]	[ ]	[ ]	[ ]	[ ]	[ ]

Periodised Carb	[ ]	[ ]	[ ]	[ ]	[ ]	[ ]	[ ]	[ ]	[ ]
FODMAP	[ ]	[ ]	[ ]	[ ]	[ ]	[ ]	[ ]	[ ]	[ ]
Other	[ ]	[ ]	[ ]	[ ]	[ ]	[ ]	[ ]	[ ]	[ ]

Comments:

What is the main purpose of following a special diet during heat training? (Tick all the answers that are important in describing what you do)*

	Cultural or religious reasons	Ethical/moral reasons	Better quality training for a special training block	Enhanced adaptation to a special training block	To lose weight/achieve body composition goals	Better health	Diagnosed allergies or intolerances	I have some allergies or intolerances but haven't had them fully diagnosed	Other (please explain)
Vegan/vegetarian	[ ]	[ ]	[ ]	[ ]	[ ]	[ ]	[ ]	[ ]	[ ]
Very high energy/extra energy (high Calorie)	[ ]	[ ]	[ ]	[ ]	[ ]	[ ]	[ ]	[ ]	[ ]
Restricted energy (low energy)	[ ]	[ ]	[ ]	[ ]	[ ]	[ ]	[ ]	[ ]	[ ]
Paleo	[ ]	[ ]	[ ]	[ ]	[ ]	[ ]	[ ]	[ ]	[ ]
Paleo for athletes	[ ]	[ ]	[ ]	[ ]	[ ]	[ ]	[ ]	[ ]	[ ]
Low Carb High Fat (LCHF)	[ ]	[ ]	[ ]	[ ]	[ ]	[ ]	[ ]	[ ]	[ ]
High Protein Low Carb	[ ]	[ ]	[ ]	[ ]	[ ]	[ ]	[ ]	[ ]	[ ]
Gluten Free	[ ]	[ ]	[ ]	[ ]	[ ]	[ ]	[ ]	[ ]	[ ]
High Carb	[ ]	[ ]	[ ]	[ ]	[ ]	[ ]	[ ]	[ ]	[ ]

Periodised Carb	[ ]	[ ]	[ ]	[ ]	[ ]	[ ]	[ ]	[ ]	[ ]
FODMAP	[ ]	[ ]	[ ]	[ ]	[ ]	[ ]	[ ]	[ ]	[ ]
Other	[ ]	[ ]	[ ]	[ ]	[ ]	[ ]	[ ]	[ ]	[ ]

Comments:

What is the main purpose of following a special diet during recovery from off-season? (Tick all the answers that are important in describing what you do)*

	Cultural or religious reasons	Ethical/moral reasons	Better quality training for a special training block	Enhanced adaptation to a special training block	To lose weight/achieve body composition goals	Better health	Diagnosed allergies or intolerances	I have some allergies or intolerances but haven't had them fully diagnosed	Other (please explain)
Vegan/vegetarian	[ ]	[ ]	[ ]	[ ]	[ ]	[ ]	[ ]	[ ]	[ ]
Very high energy/extra energy (high Calorie)	[ ]	[ ]	[ ]	[ ]	[ ]	[ ]	[ ]	[ ]	[ ]
Restricted energy (low energy)	[ ]	[ ]	[ ]	[ ]	[ ]	[ ]	[ ]	[ ]	[ ]
Paleo	[ ]	[ ]	[ ]	[ ]	[ ]	[ ]	[ ]	[ ]	[ ]
Paleo for athletes	[ ]	[ ]	[ ]	[ ]	[ ]	[ ]	[ ]	[ ]	[ ]
Low Carb High Fat (LCHF)	[ ]	[ ]	[ ]	[ ]	[ ]	[ ]	[ ]	[ ]	[ ]
High Protein Low Carb	[ ]	[ ]	[ ]	[ ]	[ ]	[ ]	[ ]	[ ]	[ ]
Gluten Free	[ ]	[ ]	[ ]	[ ]	[ ]	[ ]	[ ]	[ ]	[ ]

High Carb	[ ]	[ ]	[ ]	[ ]	[ ]	[ ]	[ ]	[ ]	[ ]
Periodised Carb	[ ]	[ ]	[ ]	[ ]	[ ]	[ ]	[ ]	[ ]	[ ]
FODMAP	[ ]	[ ]	[ ]	[ ]	[ ]	[ ]	[ ]	[ ]	[ ]
Other	[ ]	[ ]	[ ]	[ ]	[ ]	[ ]	[ ]	[ ]	[ ]

Comments:

What is the main purpose of following a special diet during recovery from injury? (Tick all the answers that are important in describing what you do)*

	Cultural or religious reasons	Ethical/moral reasons	Better quality training for a special training block	Enhanced adaptation to a special training block	To lose weight/achieve body composition goals	Better health	Diagnosed allergies or intolerances	I have some allergies or intolerances but haven't had them fully diagnosed	Other (please explain)
Vegan/vegetarian	[ ]	[ ]	[ ]	[ ]	[ ]	[ ]	[ ]	[ ]	[ ]
Very high energy/extra energy (high Calorie)	[ ]	[ ]	[ ]	[ ]	[ ]	[ ]	[ ]	[ ]	[ ]
Restricted energy (low energy)	[ ]	[ ]	[ ]	[ ]	[ ]	[ ]	[ ]	[ ]	[ ]
Paleo	[ ]	[ ]	[ ]	[ ]	[ ]	[ ]	[ ]	[ ]	[ ]
Paleo for athletes	[ ]	[ ]	[ ]	[ ]	[ ]	[ ]	[ ]	[ ]	[ ]
Low Carb High Fat (LCHF)	[ ]	[ ]	[ ]	[ ]	[ ]	[ ]	[ ]	[ ]	[ ]
High Protein Low Carb	[ ]	[ ]	[ ]	[ ]	[ ]	[ ]	[ ]	[ ]	[ ]
Gluten Free	[ ]	[ ]	[ ]	[ ]	[ ]	[ ]	[ ]	[ ]	[ ]
High Carb	[ ]	[ ]	[ ]	[ ]	[ ]	[ ]	[ ]	[ ]	[ ]

Periodised Carb	[ ]	[ ]	[ ]	[ ]	[ ]	[ ]	[ ]	[ ]	[ ]
FODMAP	[ ]	[ ]	[ ]	[ ]	[ ]	[ ]	[ ]	[ ]	[ ]
Other	[ ]	[ ]	[ ]	[ ]	[ ]	[ ]	[ ]	[ ]	[ ]

Comments:

What is the main purpose of following a special diet during preparation for competition/transition to race phase? (Tick all the answers that are important in describing what you do)*

	**Cultural or religious reasons**	**Ethical/moral reasons**	**Better quality training for a special training block**	**Enhanced adaptation to a special training block**	**To lose weight/achieve body composition goals**	**Better health**	**Diagnosed allergies or intolerances**	**I have some allergies or intolerances but haven't had them fully diagnosed**	**Other (please explain)**
Vegan/vegetarian	[ ]	[ ]	[ ]	[ ]	[ ]	[ ]	[ ]	[ ]	[ ]
Very high energy/extra energy (high Calorie)	[ ]	[ ]	[ ]	[ ]	[ ]	[ ]	[ ]	[ ]	[ ]
Restricted energy (low energy)	[ ]	[ ]	[ ]	[ ]	[ ]	[ ]	[ ]	[ ]	[ ]
Paleo	[ ]	[ ]	[ ]	[ ]	[ ]	[ ]	[ ]	[ ]	[ ]
Paleo for athletes	[ ]	[ ]	[ ]	[ ]	[ ]	[ ]	[ ]	[ ]	[ ]
Low Carb High Fat (LCHF)	[ ]	[ ]	[ ]	[ ]	[ ]	[ ]	[ ]	[ ]	[ ]
High Protein Low Carb	[ ]	[ ]	[ ]	[ ]	[ ]	[ ]	[ ]	[ ]	[ ]
Gluten Free	[ ]	[ ]	[ ]	[ ]	[ ]	[ ]	[ ]	[ ]	[ ]

High Carb	[ ]	[ ]	[ ]	[ ]	[ ]	[ ]	[ ]	[ ]	[ ]
Periodised Carb	[ ]	[ ]	[ ]	[ ]	[ ]	[ ]	[ ]	[ ]	[ ]
FODMAP	[ ]	[ ]	[ ]	[ ]	[ ]	[ ]	[ ]	[ ]	[ ]
Other	[ ]	[ ]	[ ]	[ ]	[ ]	[ ]	[ ]	[ ]	[ ]

Comments:

What is the main purpose of following a special diet during other time period? (Tick all the answers that are important in describing what you do)*

	**Cultural or religious reasons**	**Ethical/moral reasons**	**Better quality training for a special training block**	**Enhanced adaptation to a special training block**	**To lose weight/achieve body composition goals**	**Better health**	**Diagnosed allergies or intolerances**	**I have some allergies or intolerances but haven't had them fully diagnosed**	**Other (please explain)**
Vegan/vegetarian	[ ]	[ ]	[ ]	[ ]	[ ]	[ ]	[ ]	[ ]	[ ]
Very high energy/extra energy (high Calorie)	[ ]	[ ]	[ ]	[ ]	[ ]	[ ]	[ ]	[ ]	[ ]
Restricted energy (low energy)	[ ]	[ ]	[ ]	[ ]	[ ]	[ ]	[ ]	[ ]	[ ]
Paleo	[ ]	[ ]	[ ]	[ ]	[ ]	[ ]	[ ]	[ ]	[ ]
Paleo for athletes	[ ]	[ ]	[ ]	[ ]	[ ]	[ ]	[ ]	[ ]	[ ]
Low Carb High Fat (LCHF)	[ ]	[ ]	[ ]	[ ]	[ ]	[ ]	[ ]	[ ]	[ ]
High Protein Low Carb	[ ]	[ ]	[ ]	[ ]	[ ]	[ ]	[ ]	[ ]	[ ]
Gluten Free	[ ]	[ ]	[ ]	[ ]	[ ]	[ ]	[ ]	[ ]	[ ]
High Carb	[ ]	[ ]	[ ]	[ ]	[ ]	[ ]	[ ]	[ ]	[ ]

Periodised Carb	[ ]	[ ]	[ ]	[ ]	[ ]	[ ]	[ ]	[ ]	[ ]
FODMAP	[ ]	[ ]	[ ]	[ ]	[ ]	[ ]	[ ]	[ ]	[ ]
Other	[ ]	[ ]	[ ]	[ ]	[ ]	[ ]	[ ]	[ ]	[ ]

Comments:

PART B. BASE TRAINING

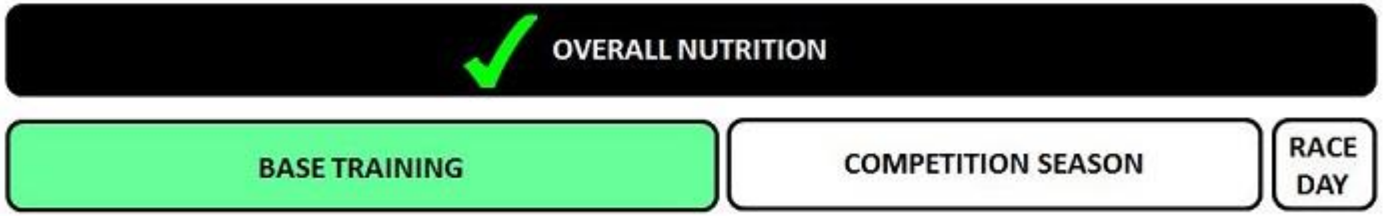

Next: Nutrition during base training (including competition preparation)

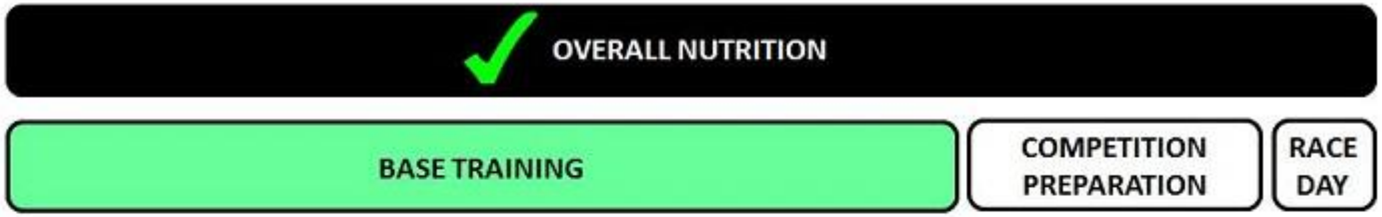

Next: Nutrition during base training

20) What is your weekly mileage (km or miles) during the base training phase (give ranges)?*

Kilometres per week: ____

Miles per week: ____

21) How many key training sessions (hard, high-intensity sessions, or long per week during this training phase (give ranges, e.g. 2-3)?

____

Focus B1. Daily eating during the base training phase

22) HARD TRAINING DAYS
Do you intentionally and purposefully eat more food/calories? [Hard training days = high volume and/or intensity days] (Tick all the answers that are important in describing what you do)*

[ ] Yes, I eat more in general

[ ] Yes, I eat more carbohydrate-rich foods

[ ] Yes, I eat more protein-rich foods

[ ] Yes, I eat more fat-rich foods or fat sources

[ ] Yes, I eat more sports foods

[ ] Yes, I eat more energy-containing drinks (e.g. protein shakes, smoothies, juice etc)

[ ] No, I do not eat more food/calories on hard training days

If yes, when do you eat more food/calories? (Tick all the answers that are important in describing what you do)

[ ] I eat more at all meals

[ ] I eat more snacks

[ ] I eat more at main meals

[ ] I eat more within 4 hours before training

[ ] I eat more during training

[ ] I eat more within 1 hour after training

[ ] I eat more just before bed

If yes, why do you eat more food/calories?

(Tick all the answers that are important in describing what you do)

[ ] I feel hungrier

[ ] I need more calories/fuel because of the higher training load

[ ] I don't want to lose weight/body fat

[ ] Other (please explain): ____*

Someone told me to eat more (who?)

( ) Coach

( ) Sports nutritionist/dietitian

( ) Physiologist

( ) Medical doctor

( ) Naturopath

( ) Physiotherapist

( ) Family member

( ) A friend

( ) Scientist

( ) Training partner

( ) I read it somewhere

( ) Personal trainer/strength&conditioning coach

If no, why do you not eat more food/calories on hard training days?

(Tick all the answers that are important in describing what you do)

[ ] I don’t feel hungrier so I don’t think I need it

[ ] I don’t feel I need more calories/fuel on hard training days

[ ] Hard training actually makes me lose my appetite so it’s hard to eat more

[ ] I am are already eating to full gut capacity so there’s no room for more

[ ] My social or financial circumstances make it hard to get access to additional food (please explain): ____*

[ ] This is an effective way to lose weight or achieve body composition goals

[ ] Other (please explain): ____*

Someone told me not to eat more (who?)

( ) Coach

( ) Sports nutritionist/dietitian

( ) Physiologist

( ) Medical doctor

( ) Naturopath

( ) Physiotherapist

( ) Family member

( ) A friend

( ) Scientist

( ) Training partner

( ) Personal trainer/Strength&conditioning coach

( ) I read it somewhere

Focus B1. Daily eating during the base training phase

23) EASY TRAINING DAYS
Do you intentionally and purposefully eat less food/calories (compared to eating in general)? (Tick all the answers that are important in describing what you do)*

[ ] Yes, I eat less in general

[ ] Yes, I eat less carbohydrate-rich foods

[ ] Yes, I eat less protein-rich foods

[ ] Yes, I eat less fat-rich foods or fat sources

[ ] Yes, I eat less sports foods

[ ] Yes, I eat less energy-containing drinks (e.g. protein shakes, smoothies, juice etc)

[ ] Yes, I eat more vegetables, salad or other low-energy foods/drinks to make up for the reduced volume of other foods

[ ] No, I do not eat less food/calories on easy training days

If yes, when do you eat less food/calories? (Tick all the answers that are important in describing what you do)

[ ] I eat less at all meals

[ ] I eat less snacks

[ ] I eat less at main meals

[ ] I eat less within 4 hours before training

[ ] I eat less during training

[ ] I eat less 1 h after training

[ ] I eat less in the evenings before bed

If yes, why do you eat less food/calories?

(Tick all the answers that are important in describing what you do)

[ ] I feel less hungry

[ ] I need fewer calories/less fuel on easy days

[ ] This is an effective strategy to help with weight/body composition goals (losing weight/body fat)

[ ] Other (please explain): ____*

Someone told me to eat less (who?)

( ) Coach

( ) Sports nutritionist/dietitian

( ) Physiologist

( ) Medical doctor

( ) Naturopath

( ) Physiotherapist

( ) Family member

( ) A friend

( ) Scientist

( ) Training partner

( ) Personal trainer/Strength&conditioning coach

( ) I read it somewhere

If no, why do you not eat less food/calories on easy training days?

(Tick all the answers that are important in describing what you do)

[ ] I still feel hungry

[ ] I need calories to fuel up for future training days

[ ] I need the calories for recovery and adaptation

[ ] I don't want to get ill or injured

[ ] I don't want to lose weight

[ ] Other (please explain): ____*

Someone told me not to eat less (who?)

( ) Coach

( ) Sports nutritionist/dietitian

( ) Physiologist

( ) Medical doctor

( ) Naturopath

( ) Physiotherapist

( ) Family member

( ) A friend

( ) Scientist

( ) Training partner

( ) Personal trainer/Strength&conditioning coach

( ) I read it somewhere

Focus B2. Timing of meals around training sessions during the base training phase

24) KEY TRAINING SESSIONS

Do you pay more attention to adequate fuelling in the 1 to 4 hours before these sessions? **[Key training session = sessions > 90 min, high quality/intensity workouts, serious gym sessions; Note: fuelling = eating foods (carbohydrates foods, protein foods, sports foods, etc.) before training]**

(Tick all the answers that are important in describing what you do)*

[ ] Yes, I eat more carbohydrate- rich foods before training

[ ] Yes, I eat more protein-rich foods before training

[ ] Yes, I eat more sports foods before training

[ ] Yes, I eat more foods in general before training

[ ] Yes, I focus on the optimal timing of my pre-training meal

[ ] No, I do not pay more attention to adequate fuelling in the 1 to 4 hours before key training sessions

If yes, why do you pay more attention to adequate fuelling?

(Tick all the answers that are important in describing what you do)

[ ] It will help me to train better at that session

[ ] I feel more energized for the whole day when I focus on fuelling before key sessions

[ ] It will reduce the risk of illness or injury

[ ] I don't want to lose weight

[ ] Other (please explain): ____*

Someone told me to pay more attention to adequate fuelling before key training sessions (who?)

( ) Coach

( ) Sports nutritionist/dietitian

( ) Physiologist

( ) Medical doctor

( ) Naturopath

( ) Physiotherapist

( ) Family member

( ) A friend

( ) Scientist

( ) Training partner

( ) Personal trainer/Strength&conditioning coach

( ) I read it somewhere

If no, why do you not pay more attention to adequate fuelling?

(Tick all the answers that are important in describing what you do)

[ ] I don’t feel it’s necessary to achieve better performance outcomes for that session

[ ] I’d like to, but eating food before higher intensity/quality sessions makes me feel sick

[ ] My finances or living arrangements make it difficult to access suitable options (please specify): ____*

[ ] My concerns around weight/body composition prevent me from eating extra foods

[ ] I haven’t received any special advice about sports nutrition

[ ] It’s difficult for me to implement any dietary changes due to my living situation (e.g. my partner/parents/roommates do most of the food preparation)

[ ] Other (please explain): ____*

Someone told me not to worry about fuelling before key training sessions (who?)

( ) Coach

( ) Sports nutritionist/dietitian

( ) Physiologist

( ) Medical doctor

( ) Naturopath

( ) Physiotherapist

( ) Family member

( ) A friend

( ) Scientist

( ) Training partner

( ) Personal trainer/Strength&conditioning coach

( ) I read it somewhere

Focus B2. Timing of meals around training sessions during the base training phase

25) KEY TRAINING SESSIONS

Do you pay more attention to adequate recovery after these sessions? [Recovery eating within 3hrs following a key session (sessions > 90 min, high quality/intensity workouts, serious gym sessions)] (Tick all the answers that are important in describing what you do)

[ ] Yes, I eat more carbohydrate-rich foods after training

[ ] Yes, I eat more sports foods after training

[ ] Yes, I eat more protein-rich foods after training

[ ] Yes, I eat more foods in general after training

[ ] Yes, I focus on timing my post-training meal within 1h after training

[ ] No, I do not pay more attention to adequate recovery after key sessions

If yes, why do you promote recovery eating?

(Tick all the answers that are important in describing what you do)

[ ] It will specifically enhance the benefits from the session I have just finished

[ ] I recover better for the whole day when I focus on recovery immediately after key sessions

[ ] It reduces the risk of illness and injury

[ ] It helps to build/retain muscle mass

[ ] I'm hungry and I feel like eating at this time

[ ] Other (please explain): ____*

Someone told me to promote recovery eating (who?)

( ) Coach

( ) Sports nutritionist/dietitian

( ) Physiologist

( ) Medical doctor

( ) Naturopath

( ) Physiotherapist

( ) Family member

( ) A friend

( ) Scientist

( ) Training partner

( ) Personal trainer/Strength&conditioning coach

( ) I read it somewhere

If no, why do you not promote recovery eating?

(Tick all the answers that are important in describing what you do)

[ ] It's not necessary for that session

[ ] I'd like to, but I have no appetite after these sessions and/or eating food at this time makes me feel sick

[ ] My finances or living arrangements make it difficult to access suitable options

[ ] My weight/body composition concerns prevent me from eating extra foods

[ ] I haven't received any special advice about sports nutrition

[ ] It’s difficult for me to implement any dietary changes due to my living situation (e.g. my partner/parents/roommates do most of the food preparation)

[ ] Other (please explain): ____*

Someone told me not to worry about recovery eating after key training sessions (who?)

( ) Coach

( ) Sports nutritionist/dietitian

( ) Physiologist

( ) Medical doctor

( ) Naturopath

( ) Physiotherapist

( ) Family member

( ) A friend

( ) Scientist

( ) Training partner

( ) Personal trainer/Strength&conditioning coach

( ) I read it somewhere

Focus B3. Training with low carbohydrate availability during the base training phase

26) Do you intentionally train in the fasted state? [Fasted training = training first thing in the morning without having eaten any food or consumed any other carbohydrates, or training later in the day without having eaten any carbohydrates for at least 8hrs prior] (Tick all the answers that are important in describing what you do)

[ ] Yes, I complete some/all of the easy training sessions in the fasted state

[ ] Yes, I complete some/all of the hard training sessions in the fasted state

[ ] No, I do not train in the fasted state

If yes, when do you train in the fasted state? (Tick all the answers that are important in describing what you do)

[ ] In the morning after an overnight fast

[ ] In the afternoon after at least 8 hours of fasting

[ ] Other (please explain): ____*

If yes, how often do you train in the fasted state?

[ ] Once a week

[ ] Twice a week

[ ] Three times a week

[ ] More than three times a week

[ ] Other (please explain): ____*

If yes, why do you train in the fasted state?

(Tick all the answers that are important in describing what you do)

[ ] I find it helps my overall training

[ ] I can't actually tell, but I believe it's supposed to help my overall training

[ ] I like to train on an empty stomach in terms of gut comfort

[ ] It means I can get up later and then go straight to training without wasting time on eating

[ ] It helps with weight loss/body composition goals

[ ] Other (please explain): ____*

Someone told me to train in the fasted state (who?)

( ) Coach

( ) Sports nutritionist/dietitian

( ) Physiologist

( ) Medical doctor

( ) Naturopath

( ) Physiotherapist

( ) Family member

( ) A friend

( ) Scientist

( ) Training partner

( ) Personal trainer/Strength&conditioning coach

( ) I read it somewhere

If yes, how long have you been doing fasted training?

[ ]

[ ] 1–3 years

[ ] 3–5 years

[ ] >5 years

If no, why do you not ever do fasted training?

(Tick all the answers that are important in describing what you do)

[ ] I feel terrible during the training session and perform so badly that it's not worth it

[ ] I find I get sick more easily

[ ] I find I get injured more easily

[ ] I do not like to train on an empty stomach/when I'm hungry

[ ] I do not feel it helps my training overall

[ ] It makes me lose weight that I don't need to lose

[ ] I don't know

[ ] This is the first time I have heard of this training / nutrition approach

[ ] Other (please explain): ____*

Someone told me not to train in the fasted state (who?)

( ) Coach

( ) Sports nutritionist/dietitian

( ) Physiologist

( ) Medical doctor

( ) Naturopath

( ) Physiotherapist

( ) Family member

( ) A friend

( ) Scientist

( ) Training partner

( ) Personal trainer/Strength&conditioning coach

( ) I read it somewhere

Focus B3. Training with low carbohydrate availability during the base training phase

27) Do you intentionally reduce your intake of carbohydrate foods periodically**?** [e.g restrict carb foods acutely or chronically during certain time periods] (Tick all the answers that are important in describing what you do)

[ ] Yes, I reduce carbohydrate intake for prolonged periods (e.g. days to weeks)

[ ] Yes, I reduce carbohydrate intake for a whole day

[ ] Yes, I reduce carbohydrate intake before easy training sessions

[ ] Yes, I reduce carbohydrate intake before hard training sessions

[ ] Yes, I reduce carbohydrate intake after easy training sessions

[ ] Yes, I reduce carbohydrate intake after hard training sessions

[ ] Yes, I reduce carbohydrate intake after the first session of the day and do the second (easy) session with low glycogen/low carb availability

[ ] Yes, I reduce carbohydrate intake after the first session of the day and do the second (hard) session with low glycogen/ low carb availability

[ ] Yes, I reduce carbohydrate intake after the second (key/hard) session of the day to sleep overnight with depleted glycogen and do the morning session with low carb availability

[ ] Yes, other (please explain): ____*

[ ] No, I do not reduce carbohydrate intake periodically

If yes, how often do you reduce carbohydrate intake during this phase?

[ ] Once a week

[ ] Twice a week

[ ] Three times a week

[ ] More than three times a week

If yes, why do you reduce carbohydrate intake periodically?

(Tick all the answers that are important in describing what you do)

[ ] I find it helps my overall training by making me adapt more

[ ] I can’t actually tell, but I believe it’s supposed to help my overall training

[ ] It is valuable for weight loss/body composition goals

[ ] I don't know

[ ] Other (please explain): ____*

Someone told me to periodically reduce carbohydrate intake (who?)

( ) Coach

( ) Sports nutritionist/dietitian

( ) Physiologist

( ) Medical doctor

( ) Naturopath

( ) Physiotherapist

( ) Family member

( ) A friend

( ) Scientist

( ) Training partner

( ) Personal trainer/Strength&conditioning coach

( ) I read it somewhere

If yes, how long have you been periodically reducing carbohydrate intake?

[ ]

[ ] 1–3 years

[ ] 3–5 years

[ ] >5 years

If no, why do you not reduce carbohydrate intake periodically?

(Tick all the answers that are important in describing what you do)

[ ] I feel terrible during the specific training session and perform so badly that it’s not worth it

[ ] I feel terrible during the whole period of training in which I am doing it, so it’s not worth it

[ ] I find I get sick more easily

[ ] I find I get injured more easily

[ ] I get hungry when I restrict carb intake

[ ] I do not feel it helps my training overall

[ ] This is the first time I have heard of this training / nutrition approach

[ ] I don't know

[ ] Other (please explain): ____*

Someone told me not to periodically reduce carbohydrate intake (who?)

( ) Coach

( ) Sports nutritionist/dietitian

( ) Physiologist

( ) Medical doctor

( ) Naturopath

( ) Physiotherapist

( ) Family member

( ) A friend

( ) Scientist

( ) Training partner

( ) Personal trainer/Strength&conditioning coach

( ) I read it somewhere

Focus B4. Training with high carbohydrate availability during the base training phase

28) Do you consume carbohydrates during workouts? (Tick all the answers that are important in describing what you do)

[ ] Yes, 1-2 times a week for key workouts

[ ] Yes, for all key workouts

[ ] Yes, 1-2 times a week, for a mixture of workouts

[ ] Yes, for all workouts, hard or easy

[ ] Yes, other (please specify): ____*

[ ] No, I do not consume carbs during workouts during the base training phase

If yes, how do you consume carbohydrates during the session? (Tick all the answers that are important in describing what you do)

[ ] I ingest carbohydrate -rich sports foods such as gels and sports drinks during training

[ ] I ingest carbohydrate -rich foods such as juice, bread, pasta, bananas or muesli bars during training

[ ] Other (please explain): ____*

If yes, why do you consume carbohydrates during the session?

(Tick all the answers that are important in describing what you do)

[ ] I find it helps me train better at that specific session

[ ] I find it helps me to practice for race fuelling strategies

[ ] I find it helps me train better for that training period

[ ] I can’t actually tell, but I believe it is supposed to help me train better

[ ] It’s part of consuming enough carbs and calories over the day when I am training hard

[ ] I don't know

[ ] Other (please explain): ____*

Someone told me to consume carbohydrates during training (who?)

( ) Coach

( ) Sports nutritionist/dietitian

( ) Physiologist

( ) Medical doctor

( ) Naturopath

( ) Physiotherapist

( ) Family member

( ) A friend

( ) Scientist

( ) Training partner

( ) Personal trainer/Strength&conditioning coach

( ) I read it somewhere

If yes, how long have you been doing this?

[ ] < 1 year

[ ] 1–3 years

[ ] 3–5 years

[ ] >5 years

If no, why do you not consume carbohydrates during workouts?

(Tick all the answers that are important in describing what you do)

[ ] I don’t think it helps my training in any way

[ ] I don’t need to practise fuelling as my race distance is so short

[ ] I’m trying to restrict carb and energy intake where possible

[ ] My stomach gets upset from eating/drinking anything during training sessions

[ ] This is the first time I have heard of this training / nutrition approach

[ ] I don't know

[ ] Other (please explain): ____*

Someone told me I don’t need to consume carbohydrates during training (who?)

( ) Coach

( ) Sports nutritionist/dietitian

( ) Physiologist

( ) Medical doctor

( ) Naturopath

( ) Physiotherapist

( ) Family member

( ) A friend

( ) Scientist

( ) Training partner

( ) Personal trainer/Strength&conditioning coach

( ) I read it somewhere

PART C. OVERALL COMPETITION SEASON (TA*) / COMPETITION PREPARATION PHASE (RA)**

* track athlete, ** road athlete

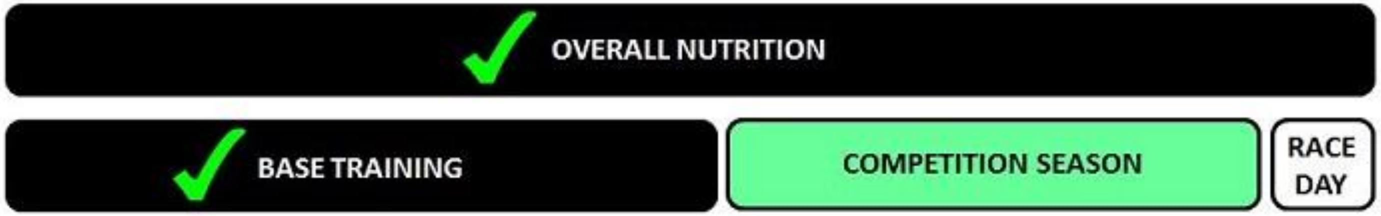

Next: Nutrition during the main competition season

General questions related to competition season (TA)

TA:29) Which of the following competition seasons is your main competition season of the year?

[ ] Winter indoor competition season (usually in February/March)

[ ] Summer outdoor competition season (usually a time period between April and September)

[ ] Fall cross-country season (usually a time period between September and December)

[ ] Other (please explain): ____*

TA:30) What is the typical length of your main competition season?*

	**Week(s)**
Length of competition season (weeks)	____

TA:31) How many serious races do you typically do within the main competition season?*

( ) 1

( ) 2

( ) 3

( ) 4

( ) 5

( ) 6

( ) 7

( ) 8

( ) 9

( ) 10

( ) More than 10

TA:32) What is your weekly mileage (km or miles) during this phase (give ranges)?*

Kilometres per week: ____

Miles per week: ____

General questions related to the preparation for competition –phase (RA)

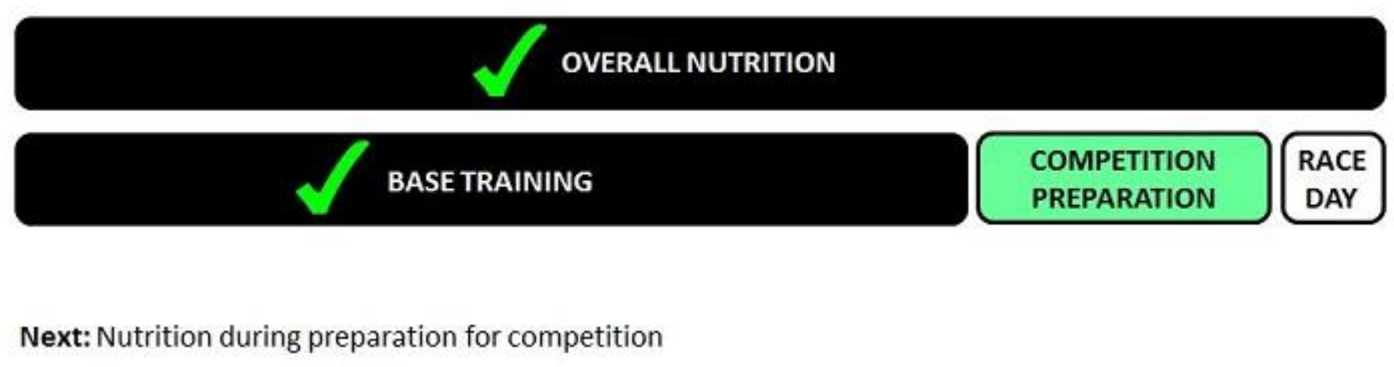

RA:33) How many weeks is the typical length of your preparation for competition period?

( ) 1

( ) 2

( ) 3

( ) 4

( ) 5

( ) 6

( ) 7

( ) 8

( ) More than 8

RA:34) How many times do you peak (for competition) per year (for example, some marathon runners might peak twice, once early in the year and the second time later in the year)?

( ) 1

( ) 2

( ) 3

( ) 4

( ) More than 4

RA:35) How many times do you typically race seriously within a peak (e.g. if you are peaking for World Championships, you might compete at both 10000m and the marathon (2 races within a peak) or 20km and 50km race walk (2 races within a peak), or just the marathon (1 race within a peak))?*

( ) 1

( ) 2

( ) 3

( ) 4

( ) More than 4

RA:36) What is your weekly mileage (km or miles) during this phase (give ranges)?*

Kilometres per week: ____

Miles per week: ____

Focus C1. Periodised nutrition during the competition phase (TA)

TA:37) Compared to your practices during base training phase (part B questions), do you change your nutrition behaviour (see below) during the competition season? [Reflect back to base training nutrition strategies and report whether your nutrition strategies change during the competition phase]
Choose the answer that best corresponds to your nutrition philosophy during the competition season (compared to base training): ***less likely to focus on a particular strategy*, *equally likely to focus on a particular strategy*, *more likely to focus on a particular strategy****

	**Less likely to focus on this strategy during the competition season**	**Equally likely to focus on this strategy during the competition season**	**More likely to focus on this strategy during the competition season**
1. Do you eat more calories on hard training days?	( )	( )	( )
2. Do you eat less calories on easy training days?:	( )	( )	( )
3. Do you focus on fuelling before key training sessions?	( )	( )	( )

4. Do you focus on recovery eating after key training sessions?	( )	( )	( )
5. Do you train in the fasted state?	( )	( )	( )
6. Do you intentionally reduce carbohydrate intake periodically?	( )	( )	( )
7. Do you ingest carbohydrates during training?	( )	( )	( )

Focus C1. Periodised nutrition during preparation for competition (RA)

RA:38) Compared to your practices during base training phase (part B questions), do you change your nutrition behaviour (see below) during preparation for competition? [Reflect back to base training nutrition strategies and report if/how your strategies change during preparation for competition]
Choose the answer that best corresponds to your nutrition philosophy during preparation for competition (compared to base training): ***less likely to focus on a particular strategy, equally likely to focus on a particular strategy, more likely to focus on a particular strategy****

	**Less likely to focus on this strategy during preparation for competition**	**Equally likely to focus on this strategy during preparation for competition**	**More likely to focus on this strategy during preparation for competition**

Do you eat more calories on hard training days?	( )	( )	( )
Do you eat less calories on easy training days?:	( )	( )	( )
Do you focus on fuelling before key training sessions?	( )	( )	( )
Do you focus on recovery eating after key training sessions?	( )	( )	( )
Do you train in the fasted state?	( )	( )	( )
Do you intentionally reduce carbohydrate intake periodically?	( )	( )	( )
Do you ingest carbohydrates during training?	( )	( )	( )

PART D. ACUTE NUTRITION IMMEDIATELY BEFORE/ON THE RACE DAY (TA & RA)

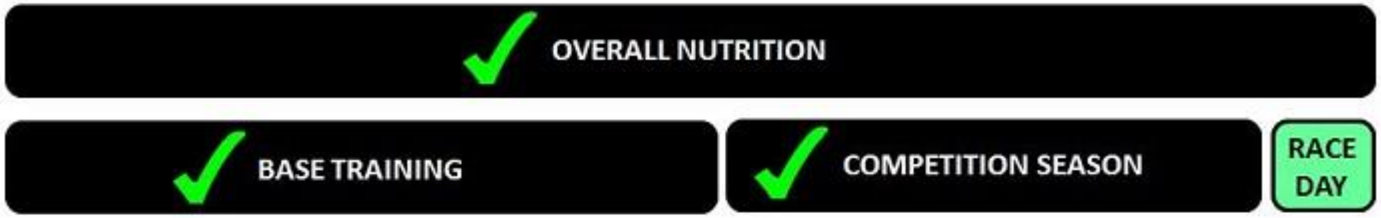

Next: Nutrition in the immediate 24-48h period around race day

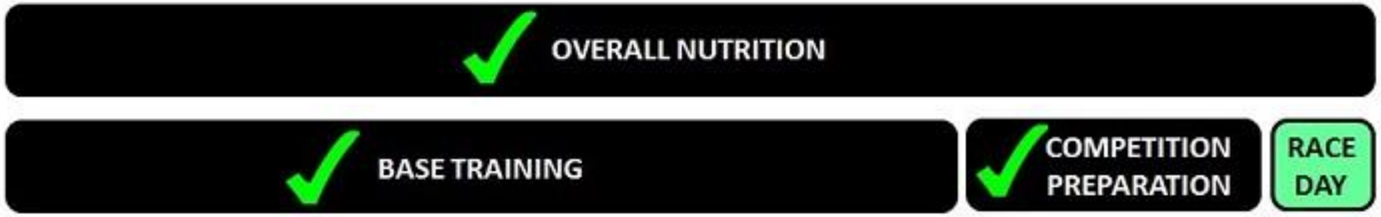

Next: Nutrition in the immediate 24-48h period around race day

Focus D1. Nutrition in the 24-48h before race day

39) Do you follow a specific diet plan in the 24-48 hours before the race day**?** (Tick all the answers that are important in describing what you do)

[ ] Yes, very high energy/extra energy (high Calorie)

[ ] Yes, restricted energy (low energy)

[ ] Yes, Low Carb High Fat (LCHF)

[ ] Yes, High Protein Low Carb

[ ] Yes, Gluten Free

[ ] Yes, High Carb/Carb loading

[ ] Yes, low residue (low fibre)

[ ] Yes, I focus on drinking more fluids

[ ] Yes, I restrict fluid intake

[ ] Yes, other (please explain): ____*

[ ] No, I do not follow any special diet plan during this period

40) If yes, why do you follow a specific dietary plan in the 24-48 hours before race day?

	I have heard it helps me perform better during the competition	I feel more energised on the competition day	I have noticed that it improves my performance on race day	I want to make sure I am adequately hydrated before the competition	I have noticed that it improves my recovery after the race	I try to avoid GI upset by excluding certain foods/ingredients	I try to get down to race weight by limiting my intake of certain foods/fluids	I am tapering	I don't know	Other (e.g. someone told you to do it: please specify in the box below)
Very high energy/extra energy (high Calorie)	[ ]	[ ]	[ ]	[ ]	[ ]	[ ]	[ ]	[ ]	[ ]	[ ]
Restricted energy (low energy)	[ ]	[ ]	[ ]	[ ]	[ ]	[ ]	[ ]	[ ]	[ ]	[ ]
Low Carb High Fat	[ ]	[ ]	[ ]	[ ]	[ ]	[ ]	[ ]	[ ]	[ ]	[ ]
High Protein Low Carb	[ ]	[ ]	[ ]	[ ]	[ ]	[ ]	[ ]	[ ]	[ ]	[ ]
Gluten Free	[ ]	[ ]	[ ]	[ ]	[ ]	[ ]	[ ]	[ ]	[ ]	[ ]
High Carb/Carb loading	[ ]	[ ]	[ ]	[ ]	[ ]	[ ]	[ ]	[ ]	[ ]	[ ]

Low residue (low fibre)	[ ]	[ ]	[ ]	[ ]	[ ]	[ ]	[ ]	[ ]	[ ]	[ ]
Focus on drinking more fluids	[ ]	[ ]	[ ]	[ ]	[ ]	[ ]	[ ]	[ ]	[ ]	[ ]
Restrict fluid intake	[ ]	[ ]	[ ]	[ ]	[ ]	[ ]	[ ]	[ ]	[ ]	[ ]
other	[ ]	[ ]	[ ]	[ ]	[ ]	[ ]	[ ]	[ ]	[ ]	[ ]

Comments:

If yes, are you usually able to follow your desired diet plan(s) for the period in the 24-48 hours before the race day?

[ ] Yes, all the time

[ ] Yes, sometimes

[ ] No, mostly not

[ ] No, never

[ ] I don’t know

What are the reasons for not being able to follow your diet plan in the 24-48 hours before the race day? (Tick all the answers that are important in describing what you do)

[ ] I am unable to bring food to the athlete village

[ ] I am unable to find appropriate foods at the race location to follow the plan

[ ] I am unable to travel with all the foods to satisfy my acute race diet

[ ] I am unable to find foods that fit my specific diet (which diet? please specify): ____*

[ ] Other (please explain): ____*

If no, why do you not follow a specific diet plan in the 24-48 hours before the race day? (Tick all the answers that are important in describing what you do)

[ ] I don’t think I need to follow any specific diet plan

[ ] I like to keep my dietary routines the same all the time

[ ] I would like to, but I am limited to foods and meal times that are available at the competition venue

[ ] I don’t know if I should follow any specific plan for my event

[ ] I don't know

[ ] Other (please explain): ____*

Focus D2. Nutrition on the race day

41) Do you follow a specific diet plan on the race day**?** (Tick all the answers that are important in describing what you do)

[ ] Yes, very high energy/extra energy (high Calorie)

[ ] Yes, restricted energy (low energy)

[ ] Yes, Low Carb High Fat (LCHF)

[ ] Yes, high protein Low Carb

[ ] Yes, Gluten Free

[ ] Yes, High Carb/Carb loading

[ ] Yes, low residue (low fibre)

[ ] Yes, I focus on drinking more fluids

[ ] Yes, I restrict fluid intake

[ ] Yes, other (please specify): ____*

[ ] No, I do not follow any special diet plan during this period

42) If yes, why do you follow a specific dietary plan on the race day?

	**I have heard it helps me perform better during the competition**	**I feel more energised on the competition day**	**I have noticed that it improves my performance on race day**	**I want to make sure I am adequately hydrated before the competition**	**I have noticed that it improves my recovery after the race**	**I try to avoid GI upset by excluding certain foods/ingredients**	**I try to get down to race weight by limiting my intake of certain foods/fluids**	**I am tapering**	**I don't know**	**Other (e.g. someone told you to do it: please specify in the box below)**
Very high energy/extra energy (high Calorie)	[ ]	[ ]	[ ]	[ ]	[ ]	[ ]	[ ]	[ ]	[ ]	[ ]
Restricted energy (low energy)	[ ]	[ ]	[ ]	[ ]	[ ]	[ ]	[ ]	[ ]	[ ]	[ ]
Low Carb High Fat	[ ]	[ ]	[ ]	[ ]	[ ]	[ ]	[ ]	[ ]	[ ]	[ ]

High Protein Low Carb	[ ]	[ ]	[ ]	[ ]	[ ]	[ ]	[ ]	[ ]	[ ]	[ ]
Gluten Free	[ ]	[ ]	[ ]	[ ]	[ ]	[ ]	[ ]	[ ]	[ ]	[ ]
High Carb/Carb loading	[ ]	[ ]	[ ]	[ ]	[ ]	[ ]	[ ]	[ ]	[ ]	[ ]
Low residue (low fibre)	[ ]	[ ]	[ ]	[ ]	[ ]	[ ]	[ ]	[ ]	[ ]	[ ]
Focus on drinking more fluids	[ ]	[ ]	[ ]	[ ]	[ ]	[ ]	[ ]	[ ]	[ ]	[ ]
Restrict fluid intake	[ ]	[ ]	[ ]	[ ]	[ ]	[ ]	[ ]	[ ]	[ ]	[ ]
Other	[ ]	[ ]	[ ]	[ ]	[ ]	[ ]	[ ]	[ ]	[ ]	[ ]

Comments:

If yes, are you usually able to follow your desired diet plan(s) for the race day?

[ ] Yes, all the time

[ ] Yes, sometimes

[ ] No, mostly not

[ ] No, never

[ ] I don’t know

What are the reasons for not being able to follow your diet plan on the race day? (Tick all the answers that are important in describing what you do)

[ ] I am unable to bring food to the athlete village

[ ] I am unable to bring my own foods/drinks to the competition venue/course or pack enough for the trip

[ ] The competition schedule sometimes changes which makes fuelling for the competition difficult

[ ] I am unable to find appropriate foods to follow the plan

[ ] I am unable to find foods that fit my specific diet (which diet?): ____*

[ ] Other (please explain): ____*

If no, why do you not follow a specific diet plan on the race day? (Tick all the answers that are important in describing what you do)

[ ] I don’t think I need to follow any specific diet plan for my event

[ ] I like to keep my dietary routines the same all the time

[ ] I would like to, but I am limited to foods and meal times that are available at the competition venue

[ ] I don’t know if I should follow any specific plan for my event

[ ] I don't know

[ ] Other (please explain): ____*

Focus D3. Nutrition during the race (RA only)

43) Do you follow a specific diet plan during the race**?** (Tick all the answers that are important in describing what you do)

[ ] Yes, I drink fluids during the race

[ ] Yes, I ingest electrolytes during the race

[ ] Yes, I ingest performance supplements (e.g. caffeine, beetroot juice) during the race (what supplements?): ____*

[ ] Yes, I ingest carbs during the race

[ ] Other (please explain): ____*

[ ] No, I do not follow any special diet plan during the race

44) If yes, why do you follow a specific dietary plan during the race?

	**I have heard it helps me perform better during the race**	**I feel more energised during the race**	**I have noticed that it improves my performance**	**I want to make sure I am adequately hydrated during the race**	**I have noticed that it improves my recovery after the race**	**I try to avoid GI upset**	**I don't know**	**Other (e.g. someone told you to do it: please specify in the box below)**
Drink fluids during the race	[ ]	[ ]	[ ]	[ ]	[ ]	[ ]	[ ]	[ ]

Ingest electrolytes during the race	[ ]	[ ]	[ ]	[ ]	[ ]	[ ]	[ ]	[ ]
Ingest performance supplements (e.g. caffeine, beetroot juice) during the race	[ ]	[ ]	[ ]	[ ]	[ ]	[ ]	[ ]	[ ]
Ingest carbs during the race	[ ]	[ ]	[ ]	[ ]	[ ]	[ ]	[ ]	[ ]
Other diet plan	[ ]	[ ]	[ ]	[ ]	[ ]	[ ]	[ ]	[ ]

Comments:

If yes, are you usually able to follow your desired diet plan(s) for during the race?

[ ] Yes, all the time

[ ] Yes, sometimes

[ ] No, mostly not

[ ] No, never

[ ] I don’t know

What are the reasons for not being able to follow your diet plan for during the race? (Tick all the answers that are important in describing what you do)

[ ] I am unable to bring my own foods/drinks to the race venue/course

[ ] The competition schedule sometimes changes which makes fuelling for the race difficult

[ ] I am unable to find appropriate foods to follow the plan

[ ] Environmental conditions make it difficult for me to follow my plan

[ ] My GI tract gets upset very easily when I'm nervous which makes me unable to follow my plan

[ ] Sometimes I miss the aid station due to running/walking in a big group of people

[ ] Other (please explain): ____*

If no, why do you not follow a specific diet plan during the race? (Tick all the answers that are important in describing what you do)

[ ] I don't think I need to follow any specific diet plan for my event

[ ] I would like to, but I am limited to aid stations available during the race

[ ] I don't know if I should follow any specific plan for my event

[ ] I don't know

[ ] Other (please explain): ____*

ADDITIONAL COMMENTS

45) OPTIONAL: If you would like to receive information on study results, please type in your email address below.

____

46) OPTIONAL: Other comments

____

____

____

____

Thank You!
